# Effects of total diet replacement programs on mental well‐being: A systematic review with meta‐analyses

**DOI:** 10.1111/obr.13465

**Published:** 2022-08-23

**Authors:** Rebecca A. Harris, Hamish A. Fernando, Radhika V. Seimon, Felipe Q. da Luz, Alice A. Gibson, Stephen W. Touyz, Amanda Sainsbury

**Affiliations:** ^1^ Faculty of Medicine and Health, Charles Perkins Centre Boden Institute of Obesity, Nutrition, Exercise & Eating Disorders, The University of Sydney Camperdown New South Wales Australia; ^2^ School of Human Sciences The University of Western Australia Crawley Western Australia Australia; ^3^ School of Biomedical Engineering, Faculty of Engineering The University of Sydney Darlington New South Wales Australia; ^4^ Faculty of Medicine, Institute of Psychiatry, Eating Disorders Program (AMBULIM) University of São Paulo São Paulo Brazil; ^5^ Menzies Centre for Health Policy and Economics, Faculty of Medicine and Health, School of Public Health The University of Sydney Camperdown New South Wales Australia; ^6^ School of Psychology, Faculty of Science The University of Sydney Camperdown New South Wales Australia

**Keywords:** diet, mental well‐being, meta‐analysis, obesity

## Abstract

This systematic review with meta‐analyses assessed the effects of total diet replacement (TDR) programs on mental well‐being in clinical trial participants with a body mass index greater than or equal to 25 kg/m^2^. TDR programs involve replacing all dietary requirements with nutritionally replete formula foods and are generally administered to induce rapid weight loss. To date, it is largely unclear what effects TDR programs may have on mental well‐being, particularly in the long‐term. To address this, we screened 25,976 references across six databases and extracted 35 publications. These 35 publications provided sufficient data to evaluate the effects of TDR programs on depression, anxiety, stress, positive affect, negative affect, vitality, role‐emotional, social functioning, mental health, mental composite summary score, self‐esteem, and general psychological health in 24 meta‐analyses. Due to the lack of research comparing TDR programs to comparator groups, 22 of our 24 meta‐analyses explored change in these mental well‐being sub‐domains over time in TDR programs without comparators. Specifically, we assessed the change from pre‐diet (before the TDR program) to either post‐diet (up to and including two months after the TDR program); and/or follow‐up (more than two months after the TDR program). For depression and anxiety, we were also able to assess the change from pre‐diet to mid‐diet (which fell within two weeks of the diet half‐way point). The remaining two meta‐analyses assessed the difference in depression scores between a TDR group and a food‐based comparator group from pre‐diet to post‐diet and from pre‐diet to follow‐up. Across all meta‐analyses, our results found no marked adverse effects of TDR programs on any mental well‐being sub‐domain. In fact, clear improvements were observed for depression, anxiety, stress, vitality, role‐emotional, and social functioning at post‐diet. Interestingly, the improvements for depression, vitality and role‐emotional were maintained at follow‐up. All improvements were observed in meta‐analyses without comparators. While the two comparator‐based meta‐analyses showed no difference between TDR programs and food‐based diets in depression symptoms, there was low statistical power. For all meta‐analyses containing three or more independent samples, we constructed prediction intervals to determine the range within which the mean of the true effects may fall for future populations. While these prediction intervals varied between sub‐domains, we found that mean depression scores are only likely to increase (i.e., depression will worsen) in less than 3% of future TDR interventions which meet our inclusion/exclusion criteria. Taken together, we concluded that for adults with a body mass index greater than or equal to 25 kg/m^2^, TDR programs are unlikely to lead to marked adverse effects on mental well‐being. These findings do not support the exclusion of participants from trials or interventions involving TDR programs based on concerns that these programs may adversely affect mental well‐being. In fact, by excluding these participants, they may be prevented from improving their metabolic health and mental well‐being.

**Abbreviations**
BMI: body mass index
SMD: standardized mean difference
TDR: total diet replacement
VLED: very low energy diet

## 1 INTRODUCTION

Obesity is a global issue linked to an increased risk of physical and psychological illness, as well as a significantly reduced quality of life.^1^, ^2^, ^3^ As weight reduction in people with obesity decreases the likelihood of developing physical and psychological illnesses,^4^, ^5^ a considerable number of obesity management strategies have been implemented with varying degrees of efficacy. One strategy, the total diet replacement (TDR) program, involves replacing all dietary requirements with nutritionally replete formula foods. TDR programs involve considerable energy restriction relative to the energy requirements for weight maintenance, and as a result, often lead to rapid weight loss. If the total energy prescription of the TDR program is equal to or below 5020 kJ (1200 kcal) per day and is equal to or above 3350 kJ (800 kcal) per day, the TDR is classified as a low energy diet (LED).^6^ If, however, the energy prescription falls below 3350 kJ (800 kcal) per day, the TDR is classified as a very low energy diet (VLED).^6^ TDR programs are often liquid in nature and are commonly utilized as the sole source of nutrition throughout an intervention. Systematic evidence supports the use of VLEDs as an effective long‐term weight loss strategy.^7^ VLEDs consistently outperform other dietary obesity treatments,^8^, ^9^, ^10^ as well as counseling weight loss strategies,^11^ in their ability to reduce excess weight in both the short‐ and long‐term in people with obesity.^12^, ^13^

While TDR programs can improve metabolic health, it is unclear what impact these diets may have on mental well‐being, particularly in people with symptoms of psychological illness. For the purposes of this review, “mental well‐being” encompasses all factors which are psychological in nature (e.g., depression, anxiety, stress, and social functioning). One branch of literature suggests that certain types of dietary energy restriction may adversely affect mental well‐being, with one noteworthy researcher coining the term “dieting depression” to describe a range of mood and behavioral symptoms experienced by participants during dietary treatments designed to reduce weight.^14^ More recently, circumstantial evidence for “dieting depression” has been noted in a retrospective population‐based study, which found that for adults over 50 years of age, depression was most likely to increase in those who lost weight over time, compared with those who either remained weight‐stable or who gained weight.^15^ However, in the above study,^15^ it is important to note that weight loss in older adults, particularly unintentional weight loss, may be indicative of an underlying health condition (e.g., dementia).^16^ Thus, the increase in depression for those who lost weight in the above study,^15^ may be linked to underlying ill health,^17^ rather than being a direct result of weight loss.

One possible mechanism by which TDR programs could reduce mental well‐being, at least in theory, is the nature of their administration. TDR programs have a limited array of meal replacement options and are, therefore, highly restrictive in choice as well as in energy prescription. Here, this restriction may heighten food cravings,^18^ and reduce the ability to alleviate negative emotional arousal through the consumption of food‐based products.^19^, ^20^ Additionally, TDR programs represent a significant shift in daily eating patterns from a predominately food‐based diet to one which is mainly liquid in nature. As there is a relationship between social interaction and food,^21^ food‐centric social engagement may be adversely affected during adherence to a TDR program, potentially occasioning an increased level of isolation and decreased mental well‐being. Finally, it is also possible that individuals with symptoms of psychological illness may be at an increased risk of exacerbated symptoms if they partake in highly restrictive and challenging diets. Individuals with depression, for example, may be prone to low compliance,^22^ and thus potential malnutrition, and/or feelings of failure, both of which could exacerbate existing mental health concerns. Some research examining the safety of LEDs and VLEDs, commonly administered using TDR programs, has found that these diets may reduce measures of mental well‐being, at least in some individuals.^6^ One randomized controlled trial, for instance, found that some participants using VLEDs reported depression, anxiety, and mood changes as side effects.^6^ Other common side effects of VLEDs, such as cold intolerance, headaches, fatigue, and joint pain,^6^, ^23^ could also indirectly lower mental well‐being.

In line with the above‐mentioned findings suggesting that certain diets may negatively influence mental well‐being, some researchers have excluded participants with pre‐existing mental health conditions from dietary trials. While one reason for such exclusions is the potential impact of mental health conditions and the associated pharmacotherapy on primary outcome variables (e.g., weight change),^24^ concerns around exacerbating mental health conditions, ^25^, ^26^ and a lack of dietary compliance,^27^ have also been referenced as reasons to exclude these participants. Serious mental disorders such as a history of or current severe psychological disturbance, psychiatric disorders, as well as unstable anxiety disorders and major depression, have all been listed as contraindications for VLED use in seminal works and national guidelines.^25^, ^28^ Interestingly, some researchers have also excluded participants with less severe mental health concerns from TDR trials. For instance, some researchers running TDR trials have excluded participants with psycho‐social problems,^29^ signs of depression,^30^ as well as participants which exhibit a psychiatric disorder of any kind,^31^ from their trials. On some occasions, no clear justification is provided for these exclusions.

While excluding people with mental health conditions is commonplace in dietary trials, several recent reports not only suggest that those with mental illness are able to successfully adhere to dietary interventions,^32^, ^33^ but also that engaging in dietary interventions can actually *improve* measures of mental well‐being.^4^, ^34^, ^35^, ^36^, ^37^, ^38^, ^39^ Interestingly, a recent systematic review with meta‐analyses which explored VLEDs,^40^ found that in comparison to before the diet, VLEDs were associated with a reduction in depression symptomatology. This change, however, was moderated by several independent factors. That is, improvements in depression were only noted when VLED protocols had the following characteristics: were longer in duration (8 to 16 weeks as opposed to 0 to 7 weeks); included low‐intensity exercise as opposed to no exercise; involved behavioral therapy; and produced a total weight loss of at least 14 kilograms. The authors of that systematic review with meta‐analyses found that in contrast to depression, anxiety levels were unaffected by VLEDs, although low power was referenced as a potential reason for that finding.^40^ In addition to the lower power referenced in that systematic review with meta‐analyses,^40^ we noticed that due in part to the widespread exclusion of participants with moderate to severe mental health concerns from TDR trials, depression and anxiety scores are often lower at baseline (indicating fewer depression and anxiety symptoms), and thus the analyses may have suffered from floor effects.

The current research, therefore, builds on this previous systematic review with meta‐analyses,^40^ by updating the literature search with more recent publications, with the intention of increasing statistical power. Additionally, we also aimed to minimize potential floor effects by conducting a broad search of several mental well‐being factors across a wide range of databases. Here, factors which are less likely to be affected by floor effects (e.g., self‐esteem and vitality) were included, and a comprehensive search was designed to capture as many participants across the mental well‐being spectrum as possible. This comprehensive literature search and subsequent analyses—including meta‐analyses, meta‐regressions and subgroup analyses—allowed us to examine many sub‐domains of mental well‐being, both before and shortly after a TDR program, as well as in the longer‐term, and to identify any factors that may moderate the relationship between TDR programs and mental well‐being. In this systematic review with meta‐analyses, we were able to evaluate the safety of TDR programs from a psychological perspective, and determine whether there is sufficient grounds to exclude participants from TDR programs based on concerns that these programs might adversely affect mental well‐being.

## 2 METHODS

The Preferred Reporting Items for Systematic Reviews and Meta‐Analyses (PRISMA) statement, updated in 2020, provided the framework for this review.^41^

### 2.1 Search strategy and information sources

Several preliminary searches were conducted across MEDLINE and Embase to trial keywords for the search strategy, and to manually extract a subset of seven publications suitable for this review. The Medical Subject Headings (MeSHs) for these seven publications were used to further refine our search terms, and through consultation with co‐authors and a meta‐analytic library specialist (T.G.), a search strategy was constructed and piloted. This search strategy was designed by coordinating search terms from the PICO (Population, Intervention, Comparison and Outcome) framework^42^, ^43^ and is shown in Table 1. Only three of the four PICO categories (Population, Intervention, and Outcome) were included in our search strategy: the Comparison category was not required for inclusion in this review, as extensive pilot testing of our search strategy revealed a lack of research examining the psychological effects of TDR programs in comparison with other diets and controls. The search strategy was further improved until it was able to detect the subset of seven publications known to be appropriate for inclusion.

**TABLE 1:** Medline search strategy

Search		Mapped and .mp (multiple field) search terms
1	Population	Limit to “humans,” limit to “all adult” Obesity, Morbid Obesity, Or Obes* or Overweight
2	Intervention	Very Low Energy Diet OR‐, VLED, Very Low Calorie Diet, VLCD, Caloric Restriction, Energy Restriction, Low Energy Diet, Liquid Diet, Weight Loss, Weight control, Diet Reducing
3	Outcome	Psychology OR‐, Psychological, Psycho‐social, Psychosocial, Depression, Anxiety, Psychological Stress, Quality of Life, Self‐concept, Self‐esteem, Self‐efficacy, Affect, Mood

Once the search strategy had been finalized, searches were conducted in November 2015 and October 2020, with a final update in January 2022, across six databases: MEDLINE, Embase, PsycINFO, CINAHL, Scopus, and Web of Science, using search terms appropriate for each database. Where possible, the “explode” function was selected to capture all associated subheadings, and the multiple field search (*.mp*) was used to extract additional publications by mapping search terms across any remaining searchable domains. Reference lists of all publications relevant to this review were manually searched for any additional publications.

### 2.2 Eligibility criteria

To be selected for this review, publications were required to be peer‐reviewed clinical trials in which only adults with a BMI of ≥25.0 kg/m^2^ were recruited (i.e., adults with a BMI in the overweight or obese range), and were subsequently administered a hypoenergetic diet (<5020 kJ [<1200 kcal] per day) with the intention of reducing weight. Only publications written in English were included in this review.

Cross‐sectional studies, literature reviews, case reports, editorials, dissertations, and study protocols were excluded, as were publications which were conducted in inpatient healthcare facilities. There was no restriction placed on participant psychological/general health status nor on the date of publication or country of origin of the research.

For publications in which BMI data was not presented^44^, ^45^ and could not be calculated from the provided data, we adopted the height/weight classification system used by the authors of these publications, to identify participants with weight in the overweight or obese categories. This system uses height and weight data to determine an expected/healthy weight for each participant. If a participant's weight exceeded this expected weight by at least 30%, the participant's weight was categorized as overweight.

The TDR program administered in all trials was required to be predominately liquid in nature, whereby meal replacement shakes and soups were used as the sole source of nutrition (albeit small amounts of vegetables, mints/gums or fiber supplements were permitted), for a period of at least 1 week in an outpatient setting. Dietary programs which were intermittent, or which specified the inclusion of meal replacement bars, and/or other food items not listed above, were excluded. Publications which did not contain any of the outcome measures specifically mentioned in our PICO search strategy in Table 1 were also excluded. Binge eating, for instance, was not included in the search strategy as the effects of severe energy restriction on binge eating have been previously explored.^46^ Thus publications which examined binge eating—but not any of the other measures listed in Table 1—were excluded.

Trial authors must have quantitatively assessed any of the outcomes of mental well‐being listed in Table 1 at a minimum of two time points; **pre‐diet** (any time prior to commencement of the TDR program); and either a **post‐diet** time point (up to and including 2 months (8.7 weeks) after completion of the TDR program); and/or a **follow‐up** time point (more than 2 months (8.7 weeks) after the TDR program), using validated measurement tools. Although not essential for this review, data corresponding to a fourth time point, **mid‐diet**, was also extracted. Mid‐diet was defined as any time point between pre‐diet and post‐diet and which fell within two weeks of the diet half‐way point.

Publications which assessed affect‐related measures through validated scales (e.g., the Beck Depression Inventory),^47^ were included in our review. As all affect‐related sub‐domains (e.g., depression) were assessed individually in this review, mental well‐being scales which combined these factors (e.g., the EQ‐5D,^48^ which assessed depression and anxiety together as a single outcome measure) were excluded. To ensure consistency within measures of mental quality of life, only generic scales which assessed mental quality of life (e.g., SF‐36),^49^ were selected for this review. That is, we did not include quality of life scales which assessed specific health concerns (e.g., sleep apnea), nor did we include weight‐specific quality of life measures (e.g., the IW‐QOL).^50^ Measurement tools which comprised both physical and mental health factors, but only provided a composite factor score, were also excluded. Additionally excluded were independent samples from trials in which bariatric surgical procedures were delivered in combination with TDR programs.

We included independent samples from trials in which weight loss pharmacotherapy was administered as part of the intervention. However, given that weight loss pharmacotherapy may affect mental well‐being,^4^ publications of trials in which pharmacotherapies were administered were subject to sensitivity analyses. As sensitivity analyses could only be conducted for our meta‐analytic data, we could not include any qualitative data drawn from publications which involved weight loss pharmacotherapies.

### 2.3 Data selection and extraction

All publications identified by the search strategy in 2015 were merged into a single library file using Endnote (Version X7, Thomson Reuters, 2013). For the searches conducted in October 2020 and in January 2022, the more recent version of Endnote (Version X9, Thomson Reuters, 2019) was used. Following the removal of publication duplicates, two authors—R.A.H. and (H.A.F. or F.Q.d.L.) screened the publication titles and abstracts and compiled a list of publications for potential inclusion in this review. Full texts for all potential publications were acquired and reviewed (library services from the University of Sydney provided publications pre‐dating electronic availability), and a final set of eligible publications was complied. Any disagreements between researchers in relation to eligibility were resolved by discussion with a third researcher (A.A.G.).

We established an internal protocol to determine which data would be selected for this review. First, for follow‐up data, our internal protocol stated that for trials which assessed a follow‐up measure at multiple time points (including trials with separate follow‐up publications), the most recent time point was selected for the follow‐up estimate. If the most recent time point was unable to be statistically assessed, the previous time point was selected. Second, for each meta‐analysis, the meta‐analytic software required us to enter the number of participants in the sample. However, if the number of participants was different at the two time points (e.g., if there had been dropouts between the pre‐diet and post‐diet measurements, and the researchers had not conducted intention‐to‐treat analyses, or we were not able to conduct intention‐to‐treat analyses ourselves from the raw dataset), we had to choose one of the two numbers (i.e., the number of participants at pre‐diet, or the [smaller] number of participants at post‐diet [completers]) for the meta‐analysis. In these cases, our internal protocol stated that we would use the number of participants at the later time point (i.e., completers). Using this smaller number of participants in the meta‐analyses—as opposed to the larger number of participants who had been measured at the earlier time point—was more conservative as it resulted in larger confidence intervals and potentially, smaller effect sizes and higher *p* values. Finally, our internal protocol stated that for trials which reported the same data in more than one publication, the publication which presented the greatest level of statistical information would be selected for review.

After the sorting and selection process, the following data was extracted from each publication: first author's family name; year of publication; participant demographics; sample size as determined above; study design; assessment scales used, measurement time points, and any methodological/statistical information required for the risk of bias assessment (see Section 2.4 for details). Statistical information necessary for the meta‐analysis (means and standard deviations) was also extracted for all mental well‐being measures at all measurement time points, as well as the means and standard deviations of the estimates of change in mental well‐being measures between time points. For consistency, standard errors were converted to standard deviations, weight data in pounds were converted to kilograms (2.2 pounds per kilogram), time data in months were converted to weeks (4.345 weeks per month), and energy data in kcal were converted to kJ (4.18 kJ per kcal).

If additional methodological and/or statistical information was required, an attempt was made to locate this information in treatment protocols and supplementary publications. Authors were contacted for any further information or data necessary for the review. Authors were also contacted when there was ambiguity over whether two publications reported the same cohort of participants. For publications in which data was not available in the necessary format for inclusion in our meta‐analyses, qualitative information was extracted and analyzed.

### 2.4 Risk of bias within publications (publication quality)

Two reviewers (R.A.H. and H.A.F.) independently rated and classified each publication for risk of bias, using a modified Downs and Black checklist.^51^ This checklist was selected as it is suitable for both randomized and non‐randomized controlled trials and has been shown to be appropriate for use in healthcare investigations.^51^, ^52^ We answered all questions which were relevant to our review. We used the overall checklist score to identify publications which may have been biased enough to warrant sensitivity analyses that included and excluded those publications. Evaluations of the four unique subscales within the checklist were used to highlight methodological/statistical strengths and weaknesses of the included publications. The checklist comprised four unique subscales, each with a different maximum score: quality of reporting (/11); external validity (/3); internal validity (/5); and selection bias (/2). These subscale scores were then summed to obtain the overall risk of bias score for the publication. A maximum score of 21 could be achieved. Articles were classified into one of the following categories to indicate overall quality: “excellent” >18; “good” 15 to 18; “fair” 11 to 14; or “poor” <11. Any disagreements were discussed until a consensus was reached.

### 2.5 Data analysis

While approximately half of all included publications (17/35) were randomized controlled trials, 11 of these 17 publications compared two or more TDR programs, and only 3 of the remaining 6 publications measured the same outcome in a non‐TDR group and could be included in a comparator‐based meta‐analysis. As over 90% of all included publications (32/35) were either non‐randomized in nature (18 publications), or randomized in nature but unable to be included in a comparator‐based meta‐analytic model (14 publications), we decided to treat most TDR groups as unique independent samples without comparators. In this way, we were able to collate the most amount of data for our review, and different mental well‐being measures drawn from the same publication were able to be included as independent samples across a range of domains and meta‐analyses. We also ran two smaller meta‐analyses to compare a TDR program to a comparator group.

**FIGURE 1:**
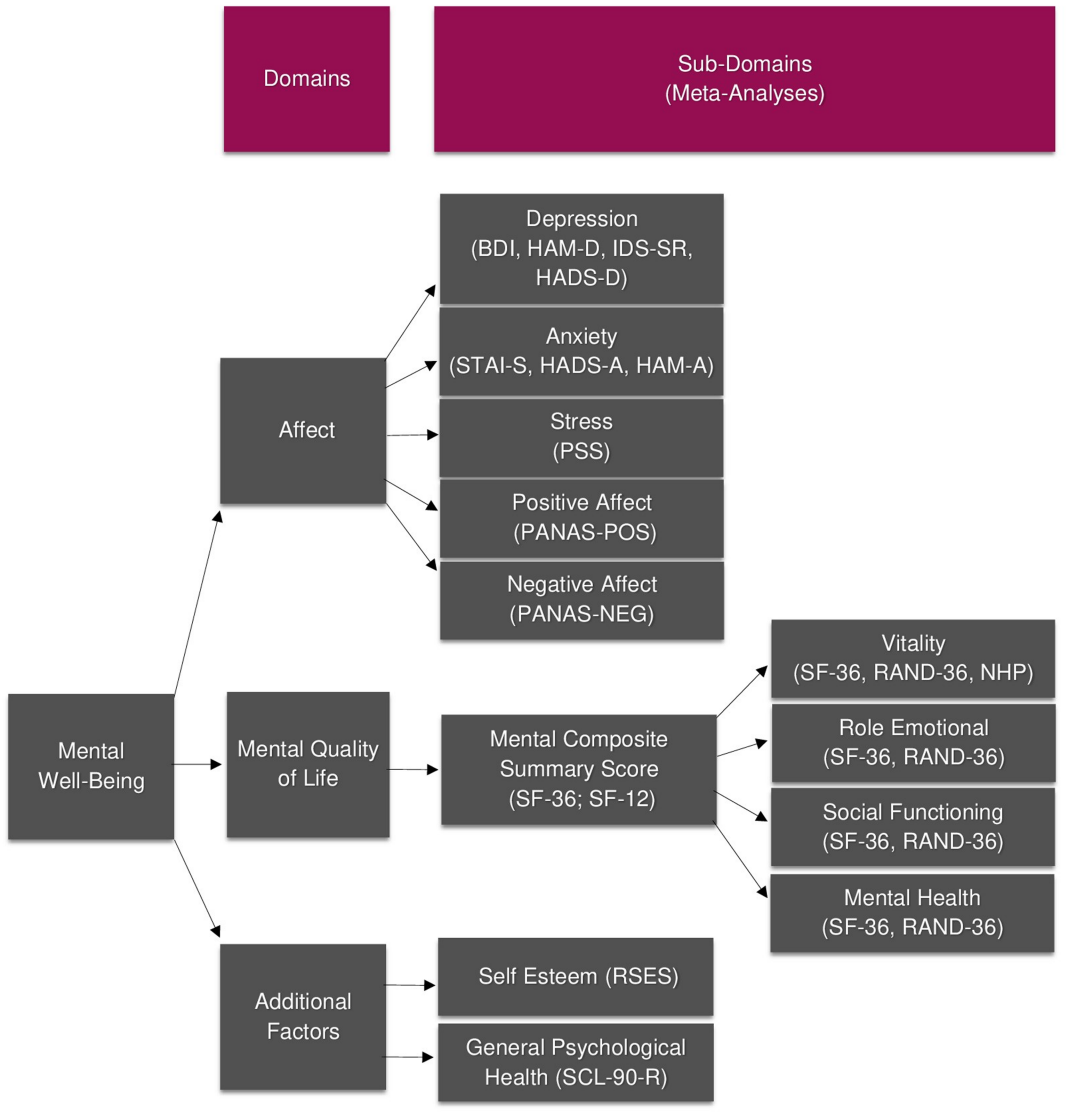
Domains, sub‐domains and measurement scales for all extracted data. Note: **BDI**, Beck Depression Inventory; **HADS‐A**, Hospital Anxiety Depression Scale (anxiety); **HADS‐D**, Hospital Anxiety Depression Scale (depression); **STAI‐S**, State Trait Anxiety Inventory (state); **HAM‐A**, Hamilton Anxiety Rating Scale; **HAM‐D**, Hamilton Depression Rating Scale; **IDS‐SR**, Inventory of Depression Symptomatology (self‐report); **NHP**, Nottingham Health Profile; **PANAS**, Positive and Negative Affect Schedule; **PSS**, Perceived Stress Scale; **RAND‐36**, RAND 36‐Item Health Survey; **RSES**, Rosenberg Self‐Esteem Scale; **SF‐12**, Short Form Health Survey with 12 Items; **SF‐36**, Short Form Health Survey with 36 Items; **SCL‐90‐R**, Symptom Checklist‐90‐Revised

Due to the wide variety of mental well‐being factors extracted from the search, data from all independent samples were organized into three main domains: **affect;** **mental quality of life;** and **additional factors** (Figure 1). **Affect** described mood‐related well‐being and incorporated five sub‐domains: depression; anxiety; stress; positive affect; and negative affect. For **mental quality of life**, the mental health scales delineated by the Short‐Form 36/Short‐Form 12 (SF‐36/SF‐12) Health Surveys,^49^ were used for our sub‐domains: vitality; role‐emotional; social functioning; and mental health—as well as a summary score of these four factors (the mental composite summary score). The **additional factors** domain of mental well‐being was composed of two sub‐domains: self‐esteem; and general psychological health. The sub‐domain of general psychological health (within the domain of additional factors) differs from the sub‐domain of mental health (within the domain of mental quality of life) in that general psychological health uses the Symptom Checklist‐90‐Revised (SCL‐90‐R)^53^ to assess somatization, obsessive–compulsive disorder, interpersonal sensitivity, depression, anxiety, hostility, phobic anxiety, paranoid ideation, and psychoticism, while the mental health sub‐domain uses the SF‐36 to examine only mood‐related factors.^49^

The effect size investigated in our meta‐analyses was change in mental well‐being between two time points (i.e., the “change estimate”), either from: pre‐diet to mid‐diet; pre‐diet to post‐diet; or pre‐diet to follow‐up. Data from an independent sample was included in a meta‐analysis if it was available in any of the following forms: (a) mean change estimate between two time points as well as the standard deviation of this change; (b) pre‐diet data (mean and standard deviation) along with either post‐diet or follow‐up data (mean and standard deviation) from which change estimates could be calculated (if available, mid‐diet data was also extracted but was not required); or (c) exact *p* values for the difference between time points, from which change estimates could also be calculated. If the exact *p* value was not provided but a significant difference between time points was reported, the most conservative *p* value (*p* = 0.05) was used to calculate change estimates for the meta‐analytic model. In the event that none of the above data types were presented in the publication, authors were contacted for additional information. Participant‐level data could be obtained for three publications.^54^, ^55^, ^56^ For these three publications,^54^, ^55^, ^56^ the “last observation carried forward” method was used to handle missing data, and change estimates were calculated using intention‐to‐treat principles.

In addition to the above data, we required the correlation between the two datasets used to calculate change estimates (with the exception of data available only as *p* values). For publications in which participant‐level data were published or available from the authors,^54^, ^55^, ^56^ correlations between outcome data at the two time points were calculated using Spearman's rho. This statistical test was selected as all participant‐level data was non‐normally distributed. The mean ± standard deviation of the correlations calculated for the participant‐level data was 0.47 ± 0.16, *n* = 40.^54^, ^55^, ^56^ For publications in which participant‐level data were not provided, and correlations between data at the two time points were not reported, a moderately‐conservative correlation estimate was assumed (*r* = 0.5).

All change estimates were standardized by expressing them as standardized mean difference (SMD) estimates. A SMD estimate represents the difference between the means of the two time points (e.g., pre‐diet mean minus post‐diet mean), divided by the pooled standard deviation of both time points. For all SMD estimates, data were arranged such that positive change estimates indicated an improvement in mental well‐being compared to before the dietary program. For each meta‐analysis, SMD estimates for all independent samples were then pooled to produce a pooled SMD estimate.

To highlight potential outliers within each meta‐analysis, the confidence interval for the SMD estimate of each independent sample was examined. If the confidence interval of an independent sample fell outside the bounds of the confidence interval for the pooled SMD estimate, this independent sample was considered a potential outlier,^57^, ^58^ and the effects of this outlier on the meta‐analysis were evaluated through sensitivity analyses. If the sensitivity analysis then determined that this independent sample exhibited a unique effect on the significance of the heterogeneity in a meta‐analysis, then this sample was omitted and the meta‐analysis was re‐run. These new meta‐analytic results were included in our review, alongside the meta‐analytic results with the independent sample included.

To account for potential heterogeneity between independent samples, a random‐effects model was used to pool SMD estimates. The assumption of this random‐effects model is that the true effect size (i.e., the effect size once sampling error has been removed) varies between independent samples.^59^ Sampling error is likely to occur as trials published in this field often have small sample sizes. Once the true effect size for each independent sample is estimated, the mean of these true effect sizes can be calculated for each meta‐analysis. The standard deviation of the true effect sizes is referred to as Tau (*T*), and the variance of the true effect sizes is referred to as Tau squared (*T* ^2^).

To understand the range within which the mean of the true effect size may fall for future populations, a prediction interval was constructed. The prediction interval predicts where the true effect size would fall for 95% of all future populations which meet the inclusion/exclusion criteria for our review.^60^

The prediction interval for each meta‐analysis was calculated as follows:

$\text{Prediction Interval}=m\pm t_{\left(\mathrm{df}\right)}\surd \left(V_{m}+T^{2}\right)$
*m* = Pooled SMD estimate of all independent samples in the meta‐analysis (random effects model)
*t*_(df)_ = Critical t value, two tailed, based on a 95% confidence interval
df = Degrees of freedom (number of independent samples in the meta‐analysis minus 2)
*V*_m_ = Variance of the pooled SMD estimate
*T* ^2^ = Tau squared (variance of the true effect sizes, as detailed above)

As we were interested in the safety of TDR programs from a psychological perspective, we used the above information to predict the probability that the SMD estimate (*m*) for future populations is ≤0 (i.e., has an adverse effect on mental well‐being).^61^ Here, a *T* score was calculated for a one‐tailed prediction to determine the probability that the pooled SMD estimate (*m*) ≤ 0. This *T* score was examined in a *T*‐distribution table to find the probability that *m* ≤ 0. As we wanted to determine the likelihood that *m* ≤ threshold “*d*,” *d* = 0 was used to represent the null effect.^61^

The *T* score was calculated as follows:

$T=\left(d–m\right)/\surd \left(V_{m}+T^{2}\right)$
*d* = 0 (Null effect)
df = Degrees of freedom (number of independent samples in the meta‐analysis minus 1)

To investigate statistical heterogeneity, we examined the Q statistic. The Q statistic provides an assessment of the total variance within each meta‐analysis which takes into account both the within‐study variance and the between‐study variance.^59^ As the Q statistic has low power with smaller samples, we set a significance threshold of *p* ≤ 0.10. If significant, this test indicates that there is significant between‐study variance in the true effect sizes (heterogeneity). It is important to note here that despite the altered *p* value, the Q statistic may still lack power if the number of independent samples and/or participants is low, and if there is substantial within‐study variance.^59^ *I* ^2^ was also calculated to represent the proportion of total variance which is attributable to between‐study variance.^60^

We established rules in our internal protocol for dealing with heterogeneity. For meta‐analyses in which the Q statistic was not significant (*p* > 0.10), no further assessment was undertaken. For meta‐analyses in which the Q statistic was significant (*p* ≤ 0.10), further investigations were conducted to determine potential sources of this between‐study variance. A series of correlations, sensitivity analyses, subgroup analyses and meta‐regressions were conducted to examine factors which we hypothesized may be contributing to this heterogeneity. To ensure that there was adequate statistical power, subgroup analyses were only conducted with meta‐analyses containing six or more independent samples, and meta‐regressions were only conducted for meta‐analyses which contained 10 or more independent samples.^62^ Each factor which was predicted to affect mental well‐being (e.g., weight change) was assessed individually in separate heterogeneity assessments.

To evaluate the potential for publication bias, Egger's test was run.^63^ As this bias testing is inappropriate for relatively small meta‐analyses due to low statistical power, these bias assessments were only undertaken for meta‐analyses with 10 or more independent samples. If significant bias was detected, sensitivity analyses and meta‐regressions were conducted as outlined above, to identify potential sources of the bias.

Comprehensive Meta‐Analysis ([computer software] version 3.3.070, 2017) was selected to conduct the above analyses^64^, and IBM SPSS Statistics ([computer software] version 27.0, 2020) was used to manage any participant‐level and supplementary data. The *p* value for all meta‐analyses, sensitivity analyses, subgroup analyses and meta‐regressions was set at ≤0.05, except for the Q statistic (*p* ≤ 0.10) as mentioned above.

## 3 RESULTS

**FIGURE 2:**
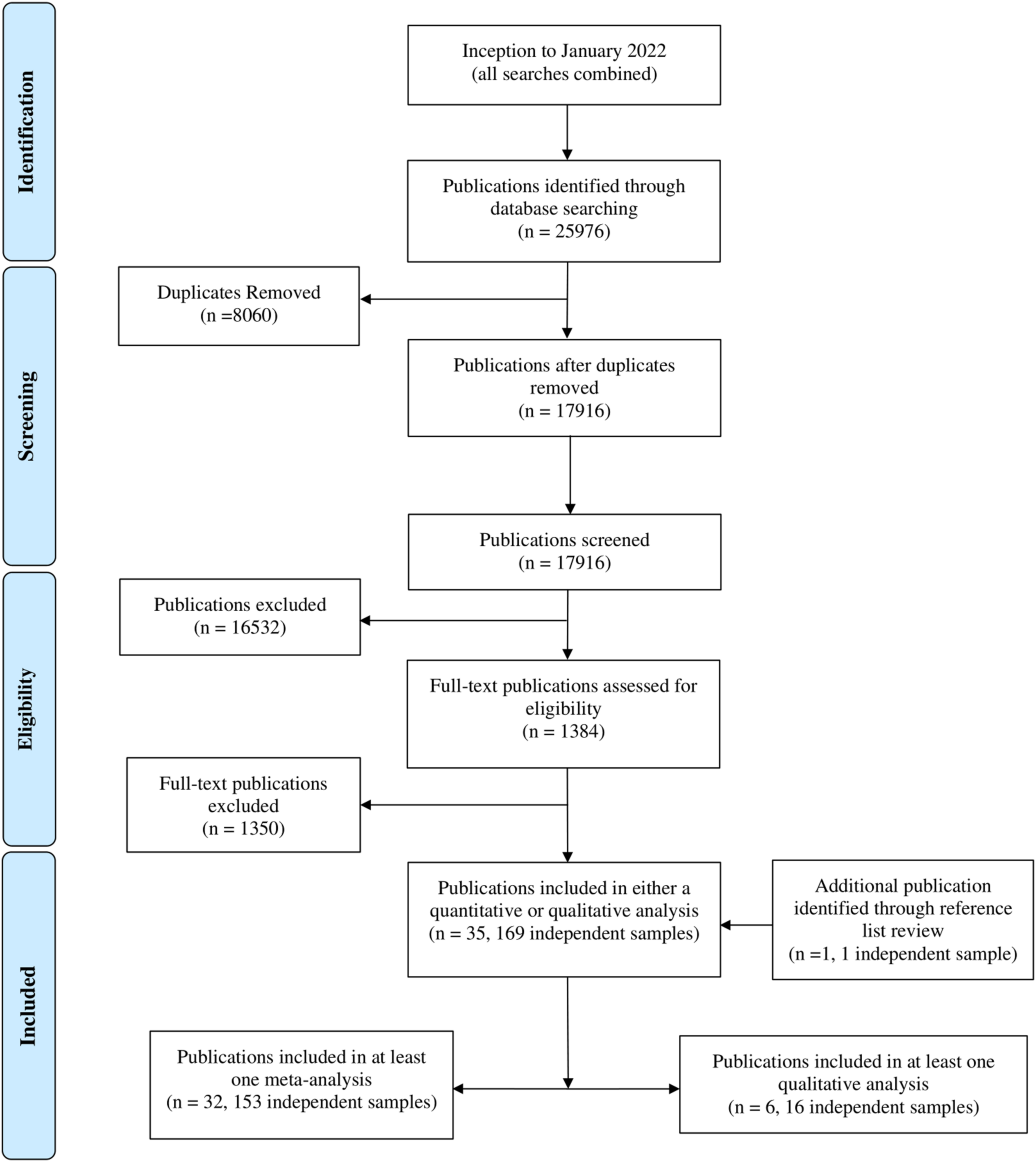
Combined totals for literature searching, screening and extraction across six databases. Note that some publications were included in at least one meta‐analysis as well as a qualitative analysis for another outcome, thus the totals do not add up to 35 publications. [Correction added on 1 September 2022, after first online publication: In the preceding sentence, the missing word ”as“ was inserted before "a qualitative".]

When combined, our literature searches yielded a total of 25,976 publications across six databases (Figure 2). Of these, 8,060 were duplicates and were removed to produce a list of 17,916 unique publications for screening. Full texts for 1384 publications were extracted and reviewed during screening, and 34 publications met all selection criteria. One additional publication was identified through reference list review, which when combined with the other included publications, produced a complete list of 35 included publications. Across these 35 publications, 169 independent samples were extracted for this review. Of these, 153 independent samples were able to be included in a meta‐analysis, while the remaining 16 independent samples were reviewed qualitatively.

Several dietary trials were close to inclusion in this review but were not included due to our inclusion/exclusion criteria. For instance, while the Diabetes Remission Clinical Trial (DiRECT)^65^, involved the administration of a liquid TDR program, the participants in that trial were assessed with the EQ‐5D scale, which only provides a score for depression and anxiety as a combination of both sub‐domains.^48^ Due to the nature of our meta‐analyses, we were only able to include trials which administered separate scales for depression and anxiety. Similarly, the Doctor Referral of Overweight People to Low Energy total diet replacement Treatment (DROPLET) trial was close to inclusion as it involved a TDR program.^66^ However, the TDR program administered in the DROPLET trial involved the consumption of meal replacement bars during the TDR phase, and was thus excluded from our review.

### 3.1 Study characteristics

Characteristics of the independent samples from publications included in this review are summarized in Tables 2A, 2B, 2C, 2D–2A, 2B, 2C, 2D. To avoid “double counting,” the following summary of independent sample characteristics excludes one publication that was included in this review,^67^ as that publication is a follow‐up of two independent samples which were also included in this review.^68^, ^69^ Due to the removal of this publication,^67^ the following section reports on 34 publications rather than the total of 35 publications which were included in our review. For one publication,^55^ while mental well‐being was assessed in a sub‐cohort of participants, demographic information was only available for the whole cohort of participants, and thus, we could only use the overall cohort demographic information in collating characteristics of the independent samples for Table 2B. Additionally, for this publication,^55^ intention to treat sample size data was used, as completers only sample size data was not available.

**TABLE 2A:** Affect: Table of publications used in our analyses of affect, listing demographic, additional method information, and statistical details

First author (year)	Country	Age ± SD (years)	BMI ± SD (range)	SS	% Fem	Psy Inc	Nut Beh Cou	Psy Cou	PA	TDR duration/method	Scale	Pre‐diet M ± SD	Mid‐diet M ± SD	Post‐diet M ± SD	Follow‐up M ± SD
Agras (1996)^b^ ^,^ ^c^	USA	43.7 ± 10.0	36.6 ± 4.4	201	100	x	✓	x	✓	12 weeks Refeeding at specified time points	BDI	7.9 ± 2.4		3.3 ± 3.3	7.3 ± 8.6
										12 weeks Refeeding when weight stable	BDI	8.1 ± 5.5		3.6 ± 5.3	6.3 ± 8.7
										12 weeks Stimulus narrowing Refeeding at specified time points	BDI	7.0 ± 5.4		2.6 ± 2.8	7.3 ± 6.6
										12 weeks Stimulus narrowing Refeeding when weight stable	BDI	8.2 ± 6.2		3.1 ± 3.2	7.2 ± 5.6
Barrett (1999)^b^	UK	42.0 ± NR	43.9 ± 7.5	115	92.2	✓	✓	x	✓	6 weeks	HADS‐D	^d^			−2.6^d^
											HADS‐A	Qualitatively reviewed No significant change in anxiety from pre‐diet to follow‐up			
Burghardt (2015)^b^	USA	51.4 ± 11.2	38.0 ± 3.4	7	100	x	x	x	✓	TDR until 15% weight reduction (mean 111 ± 20 days).	PANAS‐POS	33.0 ± 4.5		32.5 ± 7.9	
											PANAS‐NEG	10.7 ± 1.1		10.2 ± 0.4	
Burley (1992)^b^	UK	NR	34.0 (26.0 to 62.0)	8	100	✓	x	x	x	2 weeks	VAS‐mood	Qualitatively reviewed Significant reduction in irritability from Pre‐Diet to Post‐Diet (*p* < 0.05)			
Cook (1981)^b^	UK	38.0 ± 11.0	>37.0 (or at least 30 kg over ideal weight)	45	95.5	✓	x	x	x	16 weeks	BDI	14.0 ± 7.0	9.0 ± 8.0	7.0 ± 6.0	
de Zwaan (2005)^c^ ^,^ ^f^	GER	40.9 ± 7.7	36.6 ± 3.2	166	100	x	✓	✓	✓	12 weeks Participants with BED treated with CBT	BDI	10.8 ± 7.3		6.9 ± 6.9	7.5 ± 6.5
											HAM‐A	4.1 ± 4.5		3.2 ± 3.3	
		37.7 ± 6.5	35.7 ± 4.2							12 weeks Participants with BED no CBT	BDI	11.5 ± 7.9		7.1 ± 8.3	7.2 ± 8.3
											HAM‐A	4.2 ± 4.0		2.6 ± 3.0	
										12 weeks Participants without BED no CBT	BDI	7.0 ± 5.8		4.2 ± 5.5	4.9 ± 5.7
											HAM‐A	3.0 ± 4.0		2.6 ± 2.7	
										12 weeks Participants with sub‐BED no CBT	BDI	6.5 ± 5.2		4.5 ± 4.3	4.5 ± 4.3
											HAM‐A	3.5 ± 3.8		2.8 ± 3.1	
Foster (1992)^g^	USA	40.9 ± 9.6	36.7 ± 4.6	21	100	x	✓	x	✓	12 weeks 1756 kJ per day (prescribed)	BDI	8.3 ± 7.4^k^		3.4 ± 6.4^k^	See Foster (1996)
		38.8 ± 8.2	37.5 ± 5.8	23	100	x	✓	x	✓	12 weeks 2759 kJ per day (prescribed)	BDI				
		42.1 ± 10.3	38.5 ± 5.9	24	100	x	✓	x	✓	12 weeks 3344 kJ per day (prescribed)	BDI				
Foster (1996)^i^	USA	41.0 ± 9.4	39.1 ± 6.4	48	100	x	✓	x	✓	Follow‐up to Foster (1992) and Wadden (1994)	BDI	12.7 ± 8.5			9.3 ± 8.1
Herpertz (2015)^b^	GER	42.2 ± 10.8	41.2 ± 7.5	249	73.7	x	✓	x	✓	12 weeks	HADS‐D	^d^			−0.99 (−1.60 to 0.38)^d^
											HADS‐ A	^d^			+0.34 (−0.90 to 0.23)^d^
Jirik‐Babb (2003)^b^	USA	43.5 ± 9.1	31.0 ± 4.4	43	100	✓	✓	x	x	8 weeks People who binge eat	BDI	10.1 ± 6.8	6.7 ± 6.4	8.7 ± 8.2	
											BAI	6.2 ± 5.6	5.4 ± 4.2	7.9 ± 6.8	
		43.5 ± 10.0	34.6 ± 7.4							8 weeks People who do not binge eat	BDI	4.6 ± 2.7	3.5 ± 3.0	2.1 ± 1.8	
											BAI	2.5 ± 2.0	2.6 ± 2.6	2.5 ± 2.7	
Karlsson (2020)^b^	SWE	43.2 ± 12.4	42.0 ± 6.0	55	73	x	✓	✓	✓	3 months	HADS‐D	5.7 ± 4.2			5.5 ± 4.9
											HADS‐A	5.9 ± 4.2			6.2 ± 5.1
Kogon (1994)^b^ ^,^ ^g^	SWI	40.0 ± 9.6	36.5 ± 3.4	32	100	x	x	x	x	1 week (carb: fat: protein; 49%: 22%: 29%).	VAS‐Mood	Qualitatively reviewed Significant reduction in mood (worsening of symptoms) from pre‐diet to post‐diet (*p* < 0.05).			
										1 week (carb: fat: protein; 12%: 59%: 29%).	VAS‐Mood	Qualitatively reviewed No change in mood from pre‐diet to post‐diet			
Prehn (2017)^b^ ^,^ ^j^	GER	61.0 ± 6.0	34.7 ± 4.3	19	100	x	✓	x	✓	8 weeks	PANAS‐POS	33.2 ± 21.4		34.8 ± 30.6	
											PANAS‐NEG	11.1 ± 7.4		11.0 ± 4.8	
											BDI	5.8 ± 15.7		3.9 ± 15.3	
											STAI	38.8 ± 33.1		31.5 ± 32.3	
Rothberg (2015)^b^	USA	49.0 ± 8.0	41.0 ± 5.0	270	51.9	✓	✓	✓	✓	3–6 months (13.0 to 26.1 weeks)	IDS‐SR	^d^			−2.6 ± 7.0^d^
Rothberg (2016)^e^	USA	32.0 ± 4.0	41.0 ± 3.0	6	100	✓	✓	✓	✓	up to 12 weeks (TDR)	IDS‐SR	^d^		−5.0 ± 4.0^d^	
				5	100	✓	✓	✓	✓	up to 12 weeks (Food‐based control)	IDS‐SR	^d^		2.0 ± 7.0^d^	
Schrepf (2017)^**i**^	USA	50.8 ± 11.0	40.3 ± 6.5	123	66.7	✓	✓	✓	✓	up to 16 weeks	IDS‐SR	17.4 ± 9.2		11.8 ± 7.0	
Snel (2012)^b^ ^,^ ^g^	NET	53 ± 10.8	36.4 ± 4.0	13	38.5	x	x	x	✓	16 weeks	HADS‐D	3.0 ± 3.6^d^		1.0 + 0 (*p* = 0.08)^d^	2.0 ± 3.6
											HADS‐A	4.0 ± 3.6		3.0 ± 3.6	4.0 ± 3.6
	NET	56 ± 7.5	37.9 ± 5.2	14	57.1	x	x	x	x	16 weeks	HADS‐D	5.0 ± 3.7		3.0 ± 3.7	5.0 ± 3.7
											HADS‐A	5.0 ± 3.7		4.0 ± 3.7	4.0 ± 3.7
Wadden (1984)^b^	USA	37.5 ± 6.6	~39.7	17	100	✓	✓	✓	✓	1 month (4.3 weeks)	STAI‐S	^d^		−6.8^d^	−6.8^d^
											BDI	^d^		−4.6^d^	−4.6^d^
Wadden (1985)^a^ ^,^ ^g^	USA	38.1	~38.4	9	89.4	✓	✓	x	✓	4 weeks (TDR)	STAI‐S	45.9 ± 10.2	47.2 ± 12.0	38.4 ± 9.6	
											BDI	9.1 ± 7.0	8.6 ± 6.3	6.1 ± 7.5	
				7	89.4	✓	✓	x	✓	4 weeks (Food‐based control)	BDI	7.6 ± 7.8	6.7 ± 5.3	8.4 ± 1.9	
Wadden (1994)^b^	USA	36.8 ± 8.9	40.0 ± 5.7	28	100	x	✓	✓	✓	16 weeks (TDR)	BDI	13.2 ± 8.1	7.3 ± 9.3	6.7 ± 10.0	8.3 ± 9.0
				21	100	x	✓	✓	✓	16 weeks (Food‐based control)	BDI	10.1 ± 7.1	4.9 ± 5.6	5.5 ± 6.0	
Yanovski (1993)^g^ ^,^ ^b^	USA	35.8 ± 7.9	41.1 ± 7.9	8	100	x	✓	x	✓	12 weeks w. corticosteroid. People who do not binge eat	BDI	2.1 ± 3.11		1.8 ± 2.55	
		36.3 ± 6.9	41.1 ± 7.3	12	100	x	✓	x	✓	12 weeks w. corticosteroid People who binge eat	BDI	12.0 ± 9.4		4.6 ± 5.2	
Yanovski (1994)^g^	USA	36.1 ± 7.0	40.9 ± 7.4	17	100	x	✓	x	✓	12 weeks Binge eating disorder	BDI	12.7 ± 9.3			9.3 ± 8.3
		36.5 ± 7.6	39.3 ± 6.8	16	100	x	✓	x	✓	12 weeks No binge eating disorder	BDI	2.1 ± 3.1			4.8 ± 5.1

*Note*: **SD**, standard deviation, **SS**, sample size of participants (intention to treat); **% Fem**, percentage of female participants; **Psy Inc**, included participants regardless of psychological status; **Nut Beh Cou**, nutritional or behavioral counseling; **Psy Cou**, included psychological/cognitive counseling; **PA**, physical activity; **TDR**, total diet replacement; **M ± SD**, mean ± standard deviation**; NR**, not reported; **SWI**, Switzerland; **SWE**, Sweden; **UK**, United Kingdom; **USA**, United States of America; **NET**, Netherlands; **FIN**, Finland; **GER**, Germany; **BED**, binge eating disorder; **CBT**, cognitive behavioral therapy; **BDI**, Beck Depression Inventory; **BAI**, Beck Anxiety Inventory; **HADS**, Hospital Anxiety Depression Scale; **Anx**, anxiety; **Dep**, depression; **IDS‐SR**, inventory of depression symptomatology; **HAM‐A**, Hamilton Anxiety Rating Scale; **STAI‐S**, State Trait Anxiety Inventory‐State; **PANAS**, Positive and Negative Affect Schedule; **kJ per day**, kilojoules per day; ~, approximately.

^a^ Whole sample baseline demographics (including participants for which mental well‐being was not assessed).

^b^ TDR baseline demographics.

^c^ Data provided by author.

^d^ Comparison to pre‐diet.

^e^ Baseline demographics provided from completers only.

^f^ Age and BMI for study participants (not whole sample data).

^g^ Standard error of the mean was converted to standard deviation.

^i^ Baseline demographics for multiple TDR treatment groups combined.

^j^ Per protocol baseline demographics.

^k^ Statistical data collapsed across independent samples.

**TABLE 2B:** Mental quality of life: Table of publications used in our analyses of mental quality of life, listing demographic, additional method information, and statistical details

First author (year)	Country	Age ± SD (years)	BMI ± SD (range)	SS	% Fem	Psy Inc	Nut Beh Cou	Psy Cou	PA	TDR duration/ method	Scale	Sub‐domains	Pre‐diet M ± SD	Post‐diet M ± SD	Follow‐up M ± SD
Barrett (1999)^b^	UK	42.0 ± NR	43.9 ± 7.5	115	92.2	✓	✓	x	✓	6 weeks	NHP	Vitality	^d^		+31.3^d^
Bischoff (2012)^a^ ^,^ ^c^	GER	42.4 (42.2 to 42.7)	40.8 **(**40.6 to 40.9)	325	73.7	x	✓	x	✓	12 weeks	SF‐36	Vitality	46.6 ± 20.7		51.5 ± 22.7
												Mental Health	62.1 ± 20.9		66.4 ± 20.3
												Social Functioning	73.8 ± 27.5		76.9 ± 27.1
												Role‐Emotional	73.3 ± 37.4		78.0 ± 36.4
Herpetz (2015)^b^	GER	42.2 ± 10.8	41.2 ± 7.5	249	72.7	x	✓	x	✓	12 weeks	SF‐36	Mental Composite Summary Score	^d^		+1.2 ± 2.4^d^
Johansson (2011)^b^	SWE	48.7 ± 7.3	34.8 ± 2.9	63	0.0	✓	✓	x	✓	9 weeks	SF‐12	Mental Composite Summary Score	Change from pre‐diet to post‐diet +2.0 ± 2.2		
Karlsson (2020)^b^	SWE	43.2 ± 12.4	42.0 ± 6.0	55	73	x	✓	✓	✓	3 months	SF‐36	Vitality	41.6 ± 21.7		53.7 ± 24.4
												Mental Health	67.5 ± 22.9		67.1 ± 23.5
												Social Functioning	69.7 ± 30.3		64.8 ± 33.1
												Role‐Emotional	69.1 ± 41.0		66.7 ± 44.7
Kaukua (2002)^b^	FIN	45.9 ± 9.0	39.3 ± 3.3	19	0.0	✓	✓	✓	✓	10 weeks	RAND‐36 Qual reviewed	Vitality	Pre‐Diet to Post‐Diet Sig inc. Pre‐Diet to Follow‐up No Sig Diff		
												Mental Health	Pre‐Diet to Post‐Diet No Sig Diff Pre‐Diet to Follow‐up No Sig Diff		
												Social Functioning	Pre‐diet to Post‐Diet Sig inc. Pre‐diet to Follow‐up Sig inc.		
												Role‐Emotional	Pre‐Diet to Post‐Diet No Sig Diff Pre‐Diet to Follow‐up No Sig Diff		
Kaukua (2003)^b^	FIN	48.2 ± 11.1	42.8 ± 6.2	126	65.9	x	✓	✓	✓	10 weeks	RAND‐36	Vitality	^d^	Sig Inc *p* = 0.001^d^	Sig Inc *p* = 0.045^d^
												Mental Health	^d^	Sig Inc *p* = 0.001^d^	No Sig Diff *p* = 0.071^d^
												Social Functioning	^d^	Sig Inc *p* = 0.001^d^	No Sig Diff *p* = 0.069^d^
												Role‐Emotional	^d^	No Sig Diff *p* = 0.059^d^	Sig Diff *p* = 0.041^d^
Nerfeldt (2010)^b^	SWE	52 (31–68)	40 (33–50)	33	27.3	x	✓	x	✓	7 weeks Orlistat or sibutramine (*n* = 5)	SF‐36	Vitality	49.0 ± 24.0		62.0 ± 27.0
												Mental Health	74.0 ± 19.0		80.0 ± 16.0
												Social Functioning	82.0 ± 20.0		82.0 ± 25.0
												Role‐Emotional	75.0 ± 39.0		78.0 ± 36.0
OBrien (2013)^b^	AUS	53.3 ± 8.3	33.2 ± 1.3	10	60.0	x	x	x	✓	12 weeks Orlistat	SF‐36	Mental Composite Summary Score	47.7 ± 8.5		49.6 ± 5.7
Pekkarinen (2015)^c^	FIN	47.3 ± 10.5	42.1 ± 5.7	99	71.7	x	✓	✓	✓	10 weeks No after‐care maintenance.	RAND‐36	Vitality	59.0 ± 21.0	64.5 ± 22.0	59.6 ± 23.9
												Mental Health	75.6 ± 16.3	75.4 ± 18.2	72.8 ± 20.9
												Social Functioning	84.0 ± 22.4	84.2 ± 23.6	80.2 ± 26.1
												Role‐Emotional	74.5 ± 37.8	80.4 ± 33.94	70.7 ± 39.8
		47.4 ± 10.1	41.4 ± 6.4	100	71	x	✓	✓	✓	10 weeks After‐care maintenance.	RAND‐36	Vitality	64.7 ± 16.7	69.0 ± 20.1	64.4 ± 18.9
												Mental Health	76 ± 17.1	77.9 ± 17.4	76.0 ± 19.0
												Social Functioning	86.2 ± 17.4	88.0 ± 19.0	83.8 ± 21.0
												Role‐Emotional	80.4 ± 34.3	85.2 ± 30.0	81.1 ± 33.7
Snel (2012)^b^ ^,^ ^f^	NET	53 ± 10.8	36.4 ± 4.0	13	38.5	x	x	x	✓	16 weeks	NHP	Vitality	18.0 ± 25.2	2.0 ± 7.2	11.0 ± 28.8
											SF‐36	Social Functioning	81.0 ± 21.6	88.0 ± 10.8	87.0 ± 28.8
												Role‐Emotional	^d^	No Sig Diff *p* = 0.3^d^	No Sig Diff *p* = 0.3^d^
	NET	56 ± 7.5	37.9 ± 5.2	14	57.1	x	x	x	x	16 weeks	NHP	Vitality	36.0 ± 41.6	9.0 ± 18.7	31.0 ± 44.9
											SF‐36	Social Functioning	79.0 ± 18.7	88.0 ± 15.0	80.0 ± 15.0
												Role‐Emotional	74.0 ± 33.7	86.0 ± 29.9	86.0 ± 29.9
Storck (2021)^b^	GER	58.5 (53.0 to 64.0)	34.1 (32.2 to 40.6)	36	61.1	x	✓	x	✓	6 weeks	SF‐12	Mental Composite Summary Score	42.1 (36.1 to 46.7)		37.4 (30.3 to 43.7)
Subak (2005)^b^	USA	50.5 **(46 to 54)**	34 (32 to 40)	24	100	✓	✓	✓	✓	3 months (13.0 weeks)	SF‐36	Mental Composite Summary Score	50.0 ± 5.0	48.0 ± 4.0	48.0 ± 4.0
		57.5 **(50 to 62)%**	36 (32 to 38)	24	100	✓	✓	✓	✓	3 months (13.0 weeks) waitlist					
Wu (2009)^b^ ^,^ ^f^	TAI	36.6 ± 10.34	34.8 ± 4.6	33	63.6	✓	✓	x	✓	3 months (13.0 weeks)	SF‐36	Vitality	60.9 ± 19.5		71.8 ± 17.2
												Mental Health	60.6 ± 11.5		61.3 ± 10.3
												Social Functioning	80.3 ± 24.7		86.7 ± 16.1
												Role‐Emotional	71.7 ± 40.2		91.9 ± 25.3
												Mental Composite Summary Score	47.7 ± 10.9		48.8 ± 8.0

*Note*: **SD**, standard deviation; **BMI**, body mass index; **SS**, sample size of participants (intention to treat); **% Fem**, percentage of female participants; **Psy Inc**, included participants regardless of psychological status; **Nut Beh Cou**, nutritional or behavioral counseling; **Psy Cou**, included psychological/cognitive counseling; **PA**, physical activity; **TDR**, total diet replacement; **M ± SD**, mean ± standard deviation**; NR**, not reported; **PA**, physical activity; **GER**, Germany; **SWE**, Sweden; SWI, Switzerland; **UK**, United Kingdom; **USA**, United States of America; **NET**, Netherlands; **FIN**, Finland; **AUS**, Australia; **TAI**, Taiwan; **BED**, binge eating disorder; **CBT**, cognitive behavioral therapy; **VAS‐mood**, visual analogue scale; **%**Median age and interquartile range for both groups; **kj/d**, Kilojoules per day; ~, approximately; **SF‐36**, Short Form Health Survey‐36 items; **SF‐12**, Short Form Health Survey, 12 items; **RAND‐36**, The RAND 36‐Item Health Survey; **NHP**, Nottingham Health Profile; **MCS**, Mental Composite Summary Score; **Qual reviewed**, qualitatively reviewed.

^a^ Whole sample baseline demographics (including participants for which mental well‐being was not assessed).

^b^ TDR baseline demographics.

^c^ Data provided by author.

^d^ Comparison to pre‐diet.

^e^ Completers only baseline demographics.

^f^ Standard error of the mean was converted to standard deviation.

^g^ Baseline demographics for multiple TDR treatment groups combined.

^h^ Per protocol baseline demographics.

**TABLE 2C:** Self‐esteem: Table of publications used in our analyses of self‐esteem, listing demographic, additional method information, and statistical details

First author (year)	Country	Age ± SD (years)	BMI ± SD (range)	SS	% Fem	Psy Inc	Nut Beh Cou	Psy Cou	PA	TDR duration/ method	Scale	Pre‐diet M ± SD	Mid‐diet M ± SD	Post‐diet M ± SD	Follow‐up M ± SD
de Zwaan (2005)^b^ ^,^ ^c^	GER	40.9 ± 7.7	36.6 ± 3.2	166	100	x	✓	✓	✓	12 weeks Participants with BED treated with CBT	RSES	3.2 ± 0.8		3.1 ± 1.0	3.3 ± 1.0
		37.7 ± 6.5	35.7 ± 4.2							12 weeks Participants with BED not treated with CBT	RSES	3.0 ± 0.8		2.7 ± 0.7	2.8 ± 0.9
										12 weeks Participants without BED no CBT	RSES	2.8 ± 1.0		2.6 ± 1.0	2.6 ± 1.0
										12 weeks Participants with sub‐BED no CBT	RSES	2.7 ± 0.8		2.8 ± 0.8	2.8 ± 0.8
Jirik‐Babb (2003)^d^	USA	43.5 ± 9.1	31.0 ± 4.4	43	100	✓	✓	x	x	8 weeks Participants who meet binge eating criteria	RSES	Qualitatively reviewed. No significant change pre‐diet to mid‐diet			
		43.5 ± 10.0	34.6 ± 7.4							8 weeks Participants who do not meet binge eating criteria	RSES	Qualitatively reviewed. No significant change pre‐diet to mid‐diet			
Herpertz (2015)^d^	GER	42.2 ± 10.8	41.2 ± 7.5	249	72.7	x	✓	x	✓	12 weeks	RSES	^a^			+1.1^a^

*Note*: **SD**, standard deviation; **BMI**, body mass index; **SS**, sample size of participants (intention to treat); **% Fem**, percentage of female participants; **Psy Inc**, included participants regardless of psychological status; **Nut Beh Cou**, nutritional or behavioral counseling; **Psy Cou**, Included psychological/cognitive counseling; **PA**, physical activity; **TDR**, total diet replacement; **M ± SD**, mean ± standard deviation; **GER**, Germany; **USA**, United States of America; **RSES**, Rosenberg Self Esteem Scale; **BED**, binge eating disorder; **CBT**, cognitive behavioral therapy; **RSES**, Rosenberg Self‐Esteem Scale.

^a^ Change from pre‐diet.

^b^ Data provided by author.

^c^ Age and BMI for study participants (not whole sample data).

^d^ TDR baseline demographics.

**TABLE 2D:** General psychological health: Table of publications used in our analyses of general psychological health, listing demographic, additional method information, and statistical details

First author (year)	Country	Age ± SD (years)	BMI ± SD (range)	SS	% Fem	Psy Inc	Nut Beh Cou	Psy Cou	PA	TDR duration/method	Scale	Pre‐diet M ± SD	Post‐diet M ± SD	Follow‐up M ± SD
Kajaste (2004)^a^	FIN	50.1 ± 7.9	42.5 ± 4.5	17	100	x	✓	✓	✓	6 weeks (CPAP)	SCL‐90‐R (GSI)	63.7 ± 14.0		60.2 ± 17.7
		47.9 ± 8.0	45.4 ± 6.2	14	100	x	✓	✓	✓	6 weeks (non‐CPAP)	SCL‐90‐R (GSI)			
Pekkarinen (1996)^b^	FIN	41.0 (22.0 to 55.0)	36.4 (31.5 to 42.0)	62	91.9	x	✓	✓	✓	8 weeks	SCL‐90‐R (GSI)	0.36 ± 0.31	0.21 ± 0.27	0.33 ± 0.36

*Note*:  **SD**, standard deviation, **BMI**, body mass index; **SS**, sample size of participants (intention to treat); **% Fem**, percentage of female participants; **Psy Inc**, included participants regardless of psychological status; **Nut Beh Cou**, nutritional or behavioral counseling; **Psy Cou**, included psychological/cognitive counseling; **PA**, physical activity; **TDR**, total diet replacement; **M ± SD**, mean ± Standard Deviation; **FIN**, Finland; **CPAP**, continuous positive airway pressure; **SCL‐90‐R (GSI)**, Symptom Checklist‐90‐R Global Severity Index.

^a^ Per protocol baseline demographics.

^b^ TDR baseline demographics.

**TABLE 2E:** Stress: Table of the publication used in our analyses of stress, listing demographic, additional method information, and statistical details

First author (year)	Country	Age ± SD (years)	Weight ± SD (kg)	SS	% Fem	Psy Inc	Nut Beh Cou	Psy Cou	PA	TDR duration/method	Scale	Pre‐diet M ± SD	Post‐diet M ± SD
Tremblay (2021)^a^	Mul	49.8 ± 12.4	96.4 ± 22.4	138	100	x	✓	x	x	8 weeks. Women who lost less than 8% of body weight	PSS	^b^	−0.88 ± 4.91^b^
		51.7 ± 11.2	94.7 ± 18.3	1,214	100	x	✓	x	x	8 weeks. Women who lost more than or equal to 8% of body weight	PSS	^b^	−1.03 ± 5.40^b^
		49.7 ± 13.3	112.5 ± 26.1	53	0	x	✓	x	x	8 weeks. Men who lost less than 8% of body weight	PSS	^b^	−0.78 ± 5.99^b^
		53.8 ± 11.1	109.5 ± 21.1	615	0	x	✓	x	x	8 weeks. Men who lost more than or equal to 8% of body weight	PSS	^b^	−0.95 ± 5.09^b^

*Note*: **SD**, standard deviation; **BMI**, body mass index; **SS**, sample size of participants (intention to treat); **% Fem**, percentage of female participants; **Psy Inc**, included participants regardless of psychological status; **Nut Beh Cou**, nutritional or behavioral counseling; **Psy Cou**, included psychological/cognitive counseling; **PA**, physical activity; **TDR**, total diet replacement; **M ± SD**, mean ± standard deviation; **Mul**, conducted across multiple countries; **PSS**, perceived stress scale.

^a^ TDR baseline demographics.

^b^ Change from pre‐diet.

As shown in Tables 2A, 2B, 2C, 2D–2A, 2B, 2C, 2D, the trials reviewed were primarily conducted in nine countries: the United States of America (*n* = 13, providing 50 independent samples)^44^, ^45^, ^68^, ^69^, ^70^, ^71^, ^72^, ^73^, ^74^, ^75^, ^76^, ^77^, ^78^; Finland (*n* = 5, providing 35 independent samples)^56^, ^79^, ^80^, ^81^, ^82^; Germany (*n* = 5, providing 33 independent samples)^54^, ^55^, ^83^, ^84^, ^85^; the United Kingdom (*n* = 3, providing 7 independent samples)^86^, ^87^, ^88^; Sweden (*n* = 3, providing 11 independent samples)^89^, ^90^, ^91^; Switzerland (*n* = 1, providing 2 independent samples)^31^; the Netherlands (*n* = 1, providing 20 independent samples)^30^; Taiwan (*n* = 1, providing 5 independent samples)^92^; and Australia (*n* = 1, providing 1 independent sample).^29^ One trial (*n* = 1, providing 4 independent samples)^93^ was conducted across multiple countries.

While half of all 34 publications included in this review (50.0%, 17/34) were published between 1990 and 2010, 41.2% (14/34) were published between 2011 and 2021, and the remaining 8.8% (3/34) were published between 1981 and 1985. Across all 34 publications, 47.1% (16/34) were randomized controlled trials, and 58.8% (20/34) excluded participants with a history of, or with current psychiatric symptoms (Tables 2A, 2B, 2C, 2D, 2E–2A, 2B, 2C, 2D, 2E).

Across all publications, the mean participant age ranged from 32 to 61 years (median 43.5 years), while mean BMI scores were within the range of 31.0 to 45.4 kg/m^2^ (median 38.0 kg/m^2^). TDR programs were provided to participants for varying lengths of time across publications (1 to 26 weeks, with a median of 10 weeks).

Across publications, support strategies were often included in the TDR protocol. Support strategies involved behavioral/nutritional counseling, occasionally with a psychological/cognitive component, as well as recommendations to increase physical activity levels. Behavioral/nutritional counseling was administered in 79.4% (27/34) of publications, with 38.2% (13/34) of publications incorporating a psychological/cognitive counseling component. Recommendations to increase physical activity were provided in 85.3% (29/34) of all publications.

Measurement time points varied across publications whereby “pre‐diet” ranged from 1 year (52.1 weeks) to 0 weeks prior to commencing the TDR program, “mid‐diet” ranged from 2 to 8 weeks, “post‐diet” ranged from immediately upon cessation of the TDR program to up to and including 2 months (8.7 weeks) later, and “follow‐up” ranged from more than 2 months (8.7 weeks) to 8 years (417.1 weeks) after cessation of the TDR program.

### 3.2 Risk of bias within publications (publication quality)

The following section includes all 35 publications included in this review, including the aforementioned publication of a follow‐up study which was excluded from the above summary of independent sample characteristics to avoid double counting.^67^ That is because risk of bias was assessed for each publication included in the review. All 35 publications included in this review achieved a score of 11 or more out of a possible 21 on the modified Downs and Black Checklist, which represents a quality rating of “fair” or above. Overall quality scores ranged from 11 to 21, with a mean ± standard deviation of 15.7 ± 3.7 (which represents a “good” rating). The reader is reminded that the four possible quality ratings were “excellent,” “good,” “fair,” or “poor.” Specifically, 20.0% (7/35 publications) were rated as being of “fair” quality; 57.1% (20/35 publications) were rated as being of “good” quality, and 22.9% (8/35 publications) were rated as being of “excellent” quality.

**FIGURE 3:**
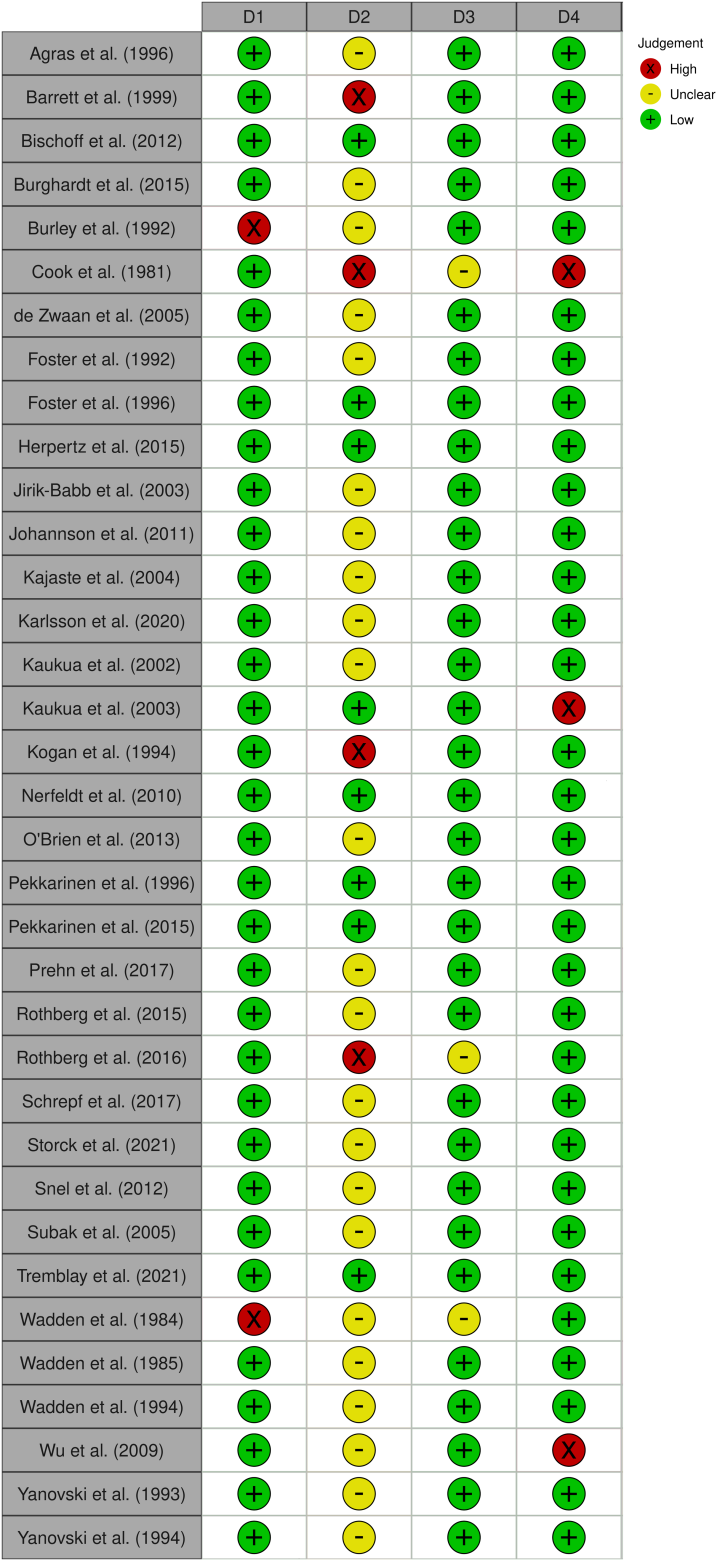
Risk of bias within publications. D1: Quality of Reporting; D2: External Validity; D3: Internal Validity; D4: Selection Bias

The results of each sub‐scale within the modified Downs and Black checklist are presented in Figure 3. Across all publications, only 5.7% (2/35 publications) were rated as having bias in quality of reporting, while only 8.6% (3/35 publications) may have been affected by bias in internal validity. Additionally, while 8.6% (3/35 publications) may have contained selection bias, the results for external validity were largely unclear, as the majority (65.7%; 23/35) of publications did not report the proportion of the source population from which the participants were derived.

Taken together, these findings suggest that while some publications may have exhibited bias in particular areas, this bias was not large enough to cause us to question the effect of any publications in this review. These findings also suggest that across all publications, the general risk of bias was low. Important to note, however, is that these quality assessments were undertaken from a modified Downs and Black checklist, and thus, the validity of this scale may have been affected.

### 3.3 Effect of total diet replacement programs on mental well‐being

A complete list of meta‐analyses is presented in Tables 3A and 3B. For Table 3A, all meta‐analyses assess change over time in independent samples without comparators, expressed as pooled SMD estimates. A positive pooled SMD estimate represents an improvement in mental well‐being from the pre‐diet value (i.e., either a reduction in depression, anxiety, stress, or negative affect scores, or an increase in positive affect, mental quality of life, self‐esteem, or general psychological health scores). Table 3B consists of two meta‐analyses which compare the difference between the TDR program and a comparator diet. Here, the SMD represents the change from pre‐diet in the TDR group minus the change from pre‐diet in the comparator group, pooled across samples. In these cases, a positive pooled SMD estimate represents greater change from pre‐diet in the TDR program compared to that in the comparator diet. Where relevant, qualitative assessments for all domains and sub‐domains of mental well‐being are presented at the end of each section below.

**TABLE 3A:** Meta‐analyses examining the effects of Total Diet Replacement programs on mental well‐being

Domain	Sub‐domain	Time points	Independent samples (participants)	Pool SMD^a^ Est	*P* value (0.05)^a^	95% confidence interval^a^	Q^b^	*P* value (0.10)^b^	*I* ^2^ ^b^	*T* ^2^ ^b^	95% prediction interval^b^	Probability of SMD ≤ 0^b^
**Affect**	**Depression**	Pre‐diet to Mid‐diet	6 (192)	0.55	**0.00**	0.36 to 0.74	2.74	0.74	0.00	0.00	0.28 to 0.81	0.11%
		Pre‐diet to Post‐diet	22 (754)	0.69	**0.00**	0.55 to 0.83	43.18	**0.03**	51.37	0.05	0.20 to 1.18	0.40%
		Pre‐diet to Follow‐up	18 (654)	0.35	**0.00**	0.14 to 0.57	106.10	**0.00**	84.00	0.17	−0.55 to 1.26	21.23%
		Pre‐diet to Follow‐up (No outlier)	17 (535)	0.28	**0.00**	0.17 to 0.38	22.14	0.14	27.74	0.01	0.04 to 0.52	2.15%
	**Anxiety**	Pre‐diet to Mid‐diet	3 (24)	−0.02	0.92	−0.42 to 0.38	0.27	0.87	0.00	0.00	−2.63 to 2.59	52.93%
		Pre‐diet to Post‐diet	11 (217)	0.27	**0.00**	0.12 to 0.42	8.78	0.55	0.00	0.00	0.09 to 0.45	0.30%
		Pre‐diet to Follow‐up	5 (184)	0.33	0.051	0.00 to 0.65	12.23	**0.02**	67.29	0.09	−0.76 to 1.42	19.72%
	**Stress**	Pre‐diet to Post‐diet	4 (2020)	0.19	**0.00**	0.14 to 0.23	0.19	**0.00**	0.00	0.00	0.10 to 0.28	0.48%
	**Positive Affect**	Pre‐diet to Post‐diet	2 (25)	0.03	0.89	−0.37 to 0.42	0.08	0.78	0.00	0.00	CBD	CBD
	**Negative Affect**	Pre‐diet to Post‐diet	2 (25)	0.13	0.54	−0.28 to 0.55	1.05	0.31	4.51	0.01	CBD	CBD
**Mental Quality of Life**	**Vitality**	Pre‐diet to Post‐diet	5 (316)	0.31	**0.00**	0.19 to 0.43	4.69	0.32	14.77	0.00	0.05 to 0.57	1.02%
		Pre‐diet to Follow‐up	10 (678)	0.23	**0.00**	0.11 to 0.35	16.20	**0.06**	44.43	0.01	−0.04 to 0.50	3.45%
	**Role‐Emotional**	Pre‐diet to Post‐diet	5 (316)	0.18	**0.00**	0.07 to 0.30	0.73	0.95	0.00	0.00	0.001 to 0.36	1.44%
		Pre‐diet to Follow‐up	9 (659)	0.13	**0.03**	0.01 to 0.25	12.76	0.12	37.30	0.01	−0.15 to 0.41	15.81%
	**Social Functioning**	Pre‐diet to Post‐diet	5 (316)	0.19	**0.03**	0.02 to 0.36	8.68	**0.07**	53.91	0.02	−0.31 to 0.68	14.58%
		Pre‐diet to Follow‐up	9 (659)	0.04	0.52	−0.08 to 0.15	13.57	**0.09**	41.04	0.01	−0.23 to 0.31	37.86%
	**Mental Health**	Pre‐diet to Post‐diet	3 (289)	0.13	0.18	−0.06 to 0.33	6.62	**0.04**	69.78	0.02	−2.06 to 2.33	26.00%
		Pre‐diet to Follow‐up	7 (599)	0.08	0.20	−0.04 to 0.21	13.00	**0.04**	53.85	0.01	−0.23 to 0.39	27.96%
	**Mental Composite Summary score** ^**c**^	Pre‐diet to Post‐diet	2 (100)	0.239	0.72	−1.08 to 1.56	34.79	**0.00**	97.13	0.88	CBD	CBD
		Pre‐diet to Follow‐up	5 (213)	−0.01	0.97	−0.47 to 0.45	35.16	**0.00**	88.62	0.23	−1.70 to 1.68	50.56%
**Additional Factors**	**Self Esteem**	Pre‐diet to Post‐diet	4 (152)	−0.11	0.26	−0.29 to 0.08	2.30	0.51	0.00	0.00	−0.53 to 0.31	65.20%
		Pre‐diet to Follow‐up	5 (271)	0.44	0.36	−0.50 to 1.39	141.55	**0.00**	97.18	1.13	−3.27 to 4.16	27.78%
	**General Psychological Health**	Pre‐diet to Follow‐up	2 (86)	0.13	0.23	−0.08 to 0.34	0.31	0.58	0.00	0.00	CBD	CBD

*Note*: **Pool SMD Est**, pooled standardized mean difference estimate; **Pre‐diet**, anytime before the diet; **Mid‐diet**, within 2 weeks of the diet midway point; **Post‐diet**, up to 2 months (8.7 weeks) after the diet; **Follow‐up**, more than 2 months (8.7 weeks) after the diet; **Q**, Q statistics; ***T***, Tau; **CBD**, cannot be determined.

^a^ Meta‐analysis statistics.

^b^ Heterogeneity statistics.

^c^ Summary score for vitality, role‐emotional, social functioning, and mental health.

**TABLE 3B:** Meta‐analyses examining comparative effects of Total Diet Replacement programs on depression against food‐based comparators

Domains	Sub‐domain	Time points	Number of comparative samples	Total TDR (participants)	Total control (participants)	Pool SMD^a^ Est	*P* value (0.05)^a^	95% confidence interval^a^	Q^b^	*P* value (0.10)^b^	*I* ^2^ ^b^	*T* ^2^ ^b^	95% prediction interval^b^	Probability of SMD ≤ 0^b^
**Affect**	**Depression**	Pre‐diet to Mid‐diet	2	37	24	0.05	0.85	−0.46 to 0.57	0.07	0.80	0.00	0.00	CBD	CBD
		Pre‐diet to Post‐diet	3	41	29	0.46	0.07	−0.03 to 0.94	2.0	0.38	0.00	0.00	−2.65 to 3.52	10.36%

*Note*: **Pool SMD Est**, pooled standardized mean difference estimate; **Pre‐diet**, anytime before the diet; **Mid‐diet**, within 2 weeks of the diet midway point; **Post‐diet**, up to 2 months (8.7 weeks) after the diet; **Follow‐up**, more than 2 months (8.7 weeks) after the diet; **Q**, Q statistics; ***T***, Tau; **CBD**, cannot be determined.

^a^ Meta‐analysis statistics.

^b^ Heterogeneity statistics.

#### 3.3.1 Affect

The reader is reminded that the domain of affect consisted of five sub‐domains (depression, anxiety, stress, positive affect, and negative affect), as shown in Figure 1 and Table 3A. Pooled SMD estimates for depression and anxiety were derived by scores from several different validated scales, while the pooled SMD estimate for stress was derived by scores from the Perceived Stress Scale (Figure 1).^94^ Positive affect and negative affect were drawn from the Positive and Negative Affect Schedule (PANAS),^95^ and meta‐analyses derived from this scale were conducted separately for positive and negative affect (PANAS‐POS and PANAS‐NEG, respectively). Positive affect described global positive mood states (i.e., attentive, active, alert, excited, enthusiastic, determined, inspired, interested, proud, and strong), while negative affect described global negative mood states (i.e., afraid, ashamed, distressed, guilty, hostile, irritable, jittery, nervous, scared, and upset). While negative affect also contained some items which may belong to the depression and anxiety sub‐domains of the domain of affect (i.e., nervous and upset), we did not separate these items from the negative affect score.

Across all sub‐domains, depression was the most frequently assessed in this review (Table 3A/3B). For one of our depression meta‐analyses (depression pre‐diet to follow‐up), a sensitivity analysis was conducted during our heterogeneity assessment, and this analysis produced the adjusted set of meta‐analytic data presented below (see Section 3.5 for details). Our final pooled SMD estimates for depression assessing change over time in independent samples without comparators found highly significant reductions in depression scores for all three time point comparisons: pre‐diet to mid‐diet; pre‐diet to post‐diet; and pre‐diet to follow‐up, with 192, 754, and 535 participants from 6, 22, and 17 independent samples, respectively (Table 3A). In our meta‐analyses, a reduction in depression scores was detected by mid‐diet (within 2 weeks of the TDR program half‐way point), and this reduction was maintained at post‐diet (up to and including 2 months (8.7 weeks) after the TDR program) and at follow‐up (more than 2 months (8.7 weeks) after the TDR program). Additionally, we found no difference between the TDR program and a food‐based comparator diet when comparing change between pre‐diet to mid‐diet (in a meta‐analysis of 2 comparative samples with a total of 61 participants from 2 publications),^45^, ^69^ nor between pre‐diet to post‐diet (in a meta‐analysis of 3 comparative samples with a total of 70 participants from 3 publications) (Table 3B).^45^, ^69^, ^77^

Further to a reduction in depression, there was a reduction in anxiety scores from pre‐diet to post‐diet (11 independent samples with 217 participants, *p* < 0.001), and there was also a trend—albeit not meeting our threshold of *p* < 0.05 for statistical significance—toward a reduction in anxiety between pre‐diet and follow‐up (5 independent samples with 184 participants, *p* = 0.051) (Table 3A). The pre‐diet to mid‐diet anxiety comparison contained fewer independent samples (*n* = 3 with 24 participants) and was non‐significant (*p* = 0.92). For our meta‐analysis of stress, we used one publication containing four independent samples and 2020 participants.^93^ We found that between pre‐diet and post‐diet, there was a significant reduction in stress (Table 3A). Taken together, these findings show that TDR programs were associated with either a reduction or no change in depression, anxiety and stress scores and that any reductions were observed by the end of the diet (post‐diet), as well as for up to 8 years (417.1 weeks) after the diet (median follow‐up was 78.2 weeks or 18 months).

The Positive and Negative Affect Schedule (PANAS) was administered in two publications,^75^, ^84^ and assessed the change in scores from pre‐diet to post‐diet. Pooled SMD estimates determined that there was no change in positive affect nor in negative affect, from pre‐diet to post‐diet (Table 3A).

Three publications assessing various sub‐domains of affect could not be added into any of the meta‐analyses presented above and thus were reviewed qualitatively.^31^, ^87^, ^88^ One publication assessed “irritability” (a sub‐component of negative affect) from pre‐diet to post‐diet in one independent sample of eight participants and found a significant reduction in symptomatology (i.e., mood improvement) (*p* < 0.05).^87^ Another publication assessed “mood” in three independent samples with a total of 32 participants to find a decrease in overall mood for one group, suggesting a worsening of symptoms, and no change in mood in the second group (the third group was excluded during our screening procedures because participants were administered a centrally‐acting serotonergic pharmacotherapy which has known effects on mood).^31^ The final publication with one independent sample of 19 participants found no significant change in anxiety from pre‐diet to follow‐up.^88^ Taken together, two of these three qualitatively‐reviewed publications found either an improvement or no change in affect, which is consistent with the results from our meta‐analyses on affect.

#### 3.3.2 Mental quality of life

The domain of mental quality of life consisted of five sub‐domains (vitality; role‐emotional; social functioning; mental health; and the mental composite summary score) (Figure 1 and Table 3A).^49^

For vitality, there was a significant increase in scores from before the diet (pre‐diet), to up to and including 2 months (8.7 weeks) after the diet (post‐diet) in a sample of 316 participants from 5 independent samples. This improvement in vitality was maintained until more than 2 months (8.7 weeks) after the diet (follow‐up), as assessed in a meta‐analysis of 10 independent samples with 678 participants. Similar improvements were observed in scores for role‐emotional (i.e., the degree to which a person can continue with their usual role—such as employment—in the face of emotional issues), as the significant improvements between pre‐diet to post‐diet in a sample of 316 participants from 5 independent samples, were maintained at follow‐up, as assessed in 659 participants from 9 independent samples.

In contrast to vitality and role‐emotional, social functioning scores showed only a transient increase (improvement) from pre‐diet to post‐diet (*p* = 0.03), in a sample of 316 participants from 5 independent samples, which was not observed at the follow‐up assessment (*p* = 0.52) with 659 participants from 9 independent samples. For mental health, no significant change was observed from pre‐diet to post‐diet, nor from pre‐diet to follow‐up, with 289 and 599 participants from 3 and 7 independent samples, respectively (Table 3A).

Six publications^29^, ^74^, ^83^, ^85^, ^90^, ^92^ containing seven independent samples, used a mental composite summary score to pool results from the above four sub‐domains (vitality, role‐emotional, social functioning, and mental health). Of these seven independent samples, the pre‐diet to post‐diet change in the mental composite summary score was examined in two independent samples with 100 participants, while the change from pre‐diet to follow‐up was examined in the remaining five independent samples with 213 participants. Interestingly, despite the majority of these four sub‐domains achieving significant increases in scores as listed above, the pooled estimates of the mental composite summary score did not achieve significance. That is, there was no significant change in the mental composite summary scores between pre‐diet and post‐diet, nor was there a significant change between pre‐diet and follow‐up. One reason for this difference in significance may be due to lack of power in the mental composite summary meta‐analyses due to the relatively low number of independent samples and participants as compared to the individual mental quality of life sub‐domains (Table 3A).

One publication,^80^ which assessed vitality, role‐emotional, social functioning, and mental health, was unable to be included in our meta‐analyses and was thus reviewed qualitatively.^80^ In that publication, the pre‐diet to post‐diet results were mostly consistent with our meta‐analyses (Table 3A), in that there were significant improvements in vitality and role‐emotional scores and no change in mental health scores. For social functioning, however, that publication reported no change from pre‐diet to post‐diet, ^80^ whereas we found a significant improvement for social functioning between pre‐diet and post‐diet. Conversely, the pre‐diet to follow‐up results for that publication^80^ were mostly inconsistent with our mental quality of life sub‐domain meta‐analyses. That is, while we found improvements in vitality and role‐emotional, and no change in social functioning at follow‐up (Table 3A), that publication^80^ found no improvements in vitality or role‐emotional and improvements in social functioning at follow‐up. Lastly, both our meta‐analysis and that publication found no change in mental health from pre‐diet to follow‐up.^80^ Taken together, the results from that qualitatively‐reviewed publication^80^ were generally consistent with our meta‐analytic results, in that the sub‐domains of mental quality of life were either improved or unchanged from pre‐diet to post‐diet and from pre‐diet to follow‐up.

#### 3.3.3 Additional factors

Self‐esteem was assessed in three publications providing 11 independent samples.^54^, ^73^, ^83^ Nine of these 11 independent samples were included in meta‐analyses, while the remaining two were included in the qualitative analysis. All of these 11 independent samples were administered the Rosenberg Self‐Esteem Scale.^96^ For self‐esteem, both the pre‐diet to post‐diet meta‐analysis (four independent samples with 152 participants), and the pre‐diet to follow‐up meta‐analysis (five independent samples with 271 participants), found no change in self‐esteem scores for either time point comparison (Table 3A). Our qualitative review of results from two independent samples with 15 participants, both drawn from one publication,^73^ similarly showed no change in self‐esteem scores between pre‐diet and mid‐diet. Taken together, there was no apparent effect of a TDR program on self‐esteem, compared to before the diet.

General psychological health was assessed in two publications,^79^, ^82^ through administration of the Symptom Checklist‐90‐Revised (SCL‐90‐R), a global scale evaluating mental well‐being by assessing a range of psychological symptoms.^53^ From these two publications,^79^, ^82^ a total of three independent samples were assessed. One independent sample with 59 participants was qualitatively reviewed.^79^ That publication found a highly significant reduction in symptomatology (*p* < 0.001) in general psychological health between pre‐diet and post‐diet, indicating a general improvement in mental well‐being.^79^ However, this improvement in general psychological health may be transient, as the other two of these three independent samples were included in a pre‐diet to follow‐up meta‐analysis with 86 participants,^79^, ^82^ and indicated no significant change in general psychological health from pre‐diet levels (Table 3A).

### 3.4 Publication bias

Publication bias testing examines the likelihood that non‐significant findings are under‐represented in the field.^97^ Four meta‐analyses contained sufficient independent samples (*n* ≥ 10) to be assessed for publication bias. Two of these meta‐analyses investigated depression (pre‐diet to post‐diet; pre‐diet to follow‐up), one assessed anxiety (pre‐diet to post‐diet) while the final meta‐analysis assessed vitality (pre‐diet to follow‐up). If publication bias is present (i.e., if non‐significant findings are underrepresented in the meta‐analysis), then the distribution of effect sizes plotted against the standard error of this estimate, is presumed to create a statistically asymmetrical funnel plot. As such, funnel plot asymmetry may indicate the presence of publication bias. Formalized testing of funnel plot asymmetry, using Egger's regression test, found that there was no evidence of asymmetry for any of these four meta‐analyses (*p* = 0.99, *p* = 0.35, *p* = 0.98, and *p* = 0.28, respectively). Taken together, these findings suggest that none of these four meta‐analyses contained significant publication bias, and as such, we can have greater confidence in the pooled SMD estimates for these meta‐analyses.

### 3.5 Heterogeneity

Across all meta‐analyses before any adjustments, *I* ^2^ (i.e., the proportion of total variance which was attributed to the between‐study variance in the true effect sizes) ranged from 0.0% to 97.2% (median 26.0%) (Table 3A). Half of all meta‐analyses (12/24) exhibited a significant Q statistic (*p* ≤ 0.10), suggesting potential heterogeneity (Tables 3A and 3B). However, only 5 of the 12 meta‐analyses (depression pre‐diet to post‐diet; depression pre‐diet to follow‐up; vitality pre‐diet to follow up; social functioning pre‐diet to follow‐up; and mental health pre‐diet to follow‐up) contained enough independent samples (six or more) for further heterogeneity investigations to be conducted, as per our internal protocol. For depression pre‐diet to follow‐up, three independent samples were not able to be included in the heterogeneity analyses due to lack of sufficient statistical information in the associated publications.^44^, ^76^, ^88^ When the observed effect sizes for depression pre‐diet to follow‐up were examined for the remaining independent samples, one independent sample^83^ was found to be an extreme outlier as the confidence interval for the SMD for this independent sample (1.35 to 1.90) fell far outside the confidence interval for the pooled SMD estimate for depression pre‐diet to follow‐up in the meta‐analysis (0.14 to 0.57). Once the meta‐analysis was adjusted by removing this outlying independent sample, the heterogeneity for depression pre‐diet to follow‐up was no longer statistically significant (*p* = 0.14, Tau^2^ = 0.01), and thus, no further analyses were conducted, as per our internal protocol outlined in Section 2 (see Table 3A for both unadjusted and adjusted depression pre‐diet to follow‐up meta‐analyses). For all four of the remaining meta‐analyses (depression pre‐diet to post‐diet; vitality pre‐diet to follow up; social functioning pre‐diet to follow‐up; and mental health pre‐diet to follow‐up), subgroup analyses were conducted, while meta‐regression analyses were also conducted for depression pre‐diet to post‐diet and for vitality pre‐diet to follow‐up, as both of these meta‐analyses contained 10 or more independent samples. Subgroup and meta‐regression analyses pertained to either participant (e.g., behavioral) or methodological factors. There was sufficient data for us to examine 11 factors in our subgroup analyses: presence (or absence) of binge eating behavior in participants; presence (or absence) of physical activity recommendations provided to participants; use (or not) of behavioral/nutritional counseling in the intervention; use (or not) of psychological/cognitive counseling in the intervention; inclusion (or exclusion) of participants based on psychological factors; type of scale used for assessment; country where the intervention was conducted; studies published prior to (or in/after) 2016, which was the year when Clinical Trial Regulations were instituted^98^; studies where the mean weight loss from pre‐diet to follow‐up was less than (or equal to/over) 5 kilograms; studies where participants had a mean pre‐diet age under (or equal to/over) 45 years; and studies where participants had a mean pre‐diet BMI under (or equal to/over) 40 kg/m^2^. For our meta‐regression analyses, there was sufficient data for us to examine nine factors, all of which were continuous variables: mean weight change; duration of the TDR program; mean pre‐diet BMI; mean age of study participants at pre‐diet; year of publication; percentage of females in the independent sample; energy content of the prescribed TDR program; study quality; and mean pre‐diet sub‐domain scores.

For our largest meta‐analysis, depression pre‐diet to post‐diet, none of the subgroup analyses provided any explanation for the heterogeneity detected. However, our meta‐regression analyses found a significant effect of year, whereby earlier publications exhibited higher SMDs indicating greater reductions in depression scores than more recent publications (Table 4A). To further our understanding of this significant effect of year on the SMD for depression pre‐diet to post‐diet, we conducted a series of bivariate correlations between pre‐diet depression score, publication year, SMD, and *p* value, using Spearman's Rho for non‐normally distributed factors. We found no significant relationship between pre‐diet depression score and publication year, nor between pre‐diet depression score and SMD. There was also no relationship between *p* value and publication year.

**TABLE 4A:** Meta‐regression examining the effect of year on standardized mean difference in the depression pre‐diet to post‐diet meta‐analysis

Domain	Sub‐domain	Time points	Factor	Independent samples	Coefficient	Standard error	95% confidence interval	*R* ^2^	*P* value*
**Affect**	Depression	Pre‐diet to Post‐diet	Year	22	−0.014	0.001	−0.03 to −0.00	0.33	0.049

* Threshold at 0.05.

For vitality pre‐diet to follow‐up, two factors emerged significant in our subgroup analyses. First, we found that while independent samples with an average weight loss of less than 5 kilograms at follow‐up showed no improvements in vitality scores, independent samples with an average weight loss of 5 kilograms or more at follow‐up showed improvements in vitality scores (Table 4B). Second, we found that independent samples which comprised participants with an average age younger than 45 years at pre‐diet showed significant improvements in vitality scores, whereas improvements in vitality between pre‐diet and follow‐up were not observed for independent samples which comprised participants with an average age of equal to or older than 45 years at pre‐diet (Table 4B).

For mental health pre‐diet to follow‐up, our subgroup analyses found—paradoxically—that independent samples that included psychological/cognitive counseling in the intervention exhibited no change in mental health scores from pre‐diet to follow‐up, while independent samples that did not include psychological/cognitive counseling in the intervention showed significant improvements in scores over this time frame (Table 4B).

**TABLE 4B:** Subgroups analyses examining the effect of mediating factors on the vitality and mental health sub‐domains

Domain	Sub‐domain	Time points	Factor	Independent samples	Pooled SMD estimate	Standard error	95% confidence interval	*P* value*
**Mental Quality of Life**	Vitality	Pre‐diet to Follow‐up^a^	Average weight loss less than 5 kg at follow‐up	3	0.07	0.07	−0.07 to 0.22	0.002
			Average weight loss more than 5 kg at follow‐up	6	0.45	0.10	0.26 to 0.64	
			Average age younger than 45 years old at pre‐diet	4	0.39	0.11	0.17 to 0.61	0.033
			Average age older than 45 years old at pre‐diet	6	0.11	0.07	−0.01 to 0.24	
	Mental Health	Pre‐diet to Follow‐up	Cognitive training provided	4	0.01	0.08	−0.14 to 0.16	0.032
			Cognitive training not provided	3	0.20	0.05	0.11 to 0.30	

*Note*: **Pooled SMD Estimate**, pooled standardized mean difference estimate; **BMI**, body mass index.

^a^ Bischoff et al. (2012) was not included in this subgroup analysis due to lack of available data.

* Threshold at 0.05 for comparison between subgroups.

For social functioning pre‐diet to follow‐up, none of the 11 factors included in our subgroup analyses could explain the significant heterogeneity in this meta‐analysis (data not shown).

In sum, while some moderating factors (i.e., publication year as a continuous variable; whether mean weight loss from pre‐diet to follow‐up was less than (or equal to/greater than) 5 kilograms; whether mean age at pre‐diet was less than (or equal to/greater than) 45 years; whether participants had a mean pre‐diet BMI under (or equal to/over) 40 kg/m^2^; and use (or not) of psychological/cognitive counseling in the intervention) exhibited significant effects on the meta‐analyses listed above, the majority of the potential moderating factors investigated (e.g., use (or not) of behavioral/nutritional counseling in the intervention; studies published prior to (or in/after) 2016, which was the year when Clinical Trial Regulations were instituted^98^; as well as the inclusion (or exclusion) of participants based on psychological factors) exhibited no effect on the results in any of our heterogeneity assessments.

### 3.6 Prediction intervals

Prediction intervals were constructed to determine the likelihood that future TDR interventions which meet our inclusion/exclusion criteria may adversely affect mental well‐being, either by increasing depression, anxiety, stress or negative affect scores, or by decreasing mental quality of life or self‐esteem scores. Across the mental well‐being sub‐domains examined in this review, sufficient data was available to construct prediction intervals for depression, anxiety, stress, all of the mental quality of life sub‐domains, as well as the mental composite summary score, and self‐esteem. For the meta‐analyses of depression pre‐diet to mid‐diet, pre‐diet to post‐diet, and pre‐diet to follow‐up (no outlier included) without comparators, the probability that future TDR interventions will exhibit an increase in mean depression scores from mean pre‐diet levels were 0.11%, 0.40%, and 2.15%, respectively (Table 3A). Interestingly, while the depression pre‐diet to post‐diet meta‐analysis which included a dietary food‐based comparator found no significant difference between the TDR and comparator groups in their respective changes from pre‐diet over time (Table 3B), the prediction interval found that for future TDR trials which include food‐based comparator groups, only 10.36% of these trials are likely to show greater improvement in depression scores for the food‐based comparator group when compared with the TDR group.^61^ Prediction intervals also determined that for anxiety pre‐diet to post‐diet, and pre‐diet to follow‐up, only 0.30% and 19.72% of future interventions involving TDR programs with the same inclusion/exclusion used for this review, are likely to exhibit an increase in mean anxiety scores. For anxiety pre‐diet to mid‐diet, although the prediction interval found that 52.93% of future interventions involving TDR programs may exhibit an increase in mean anxiety scores from pre‐diet levels, the power of this prediction interval was low, with only 3 independent samples and 24 participants. Additionally, the prediction interval for stress determined that only 0.48% of future trials examining stress are likely to report a mean increase in stress levels between pre‐diet and post‐diet. For the four mental quality of life sub‐domains (vitality, role‐emotional, social functioning, and mental health), prediction intervals ranged from 1.02% to 37.86% (median 14.58%). Interestingly, despite this range, the prediction interval for the mental composite summary score (a pooled summary of the four sub‐domains of mental quality of life) for pre‐diet to follow‐up was 50.56%. Again, this may be due to low power, as all four mental quality of life sub‐domains contained more independent samples and/or participants than the meta‐analyses for the mental composite summary score (Table 3A). Finally, the prediction interval for self‐esteem from pre‐diet to post‐diet, containing 4 independent samples with 152 participants (all of which were drawn from one publication)^54^ showed that up to 65.20% of future trials involving TDR interventions may exhibit a decrease in self‐esteem from pre‐diet to post‐diet, although this was reduced to 27.78% by follow‐up (5 independent samples with 271 participants).

### 3.7 Sensitivity analyses for centrally‐ and peripherally‐acting pharmacotherapies

Centrally‐ and peripherally‐acting pharmacotherapies were administered in a total of eight independent samples drawn from four publications.^29^, ^67^, ^70^, ^89^ These pharmacotherapies were administered either before,^70^ or after,^29^, ^67^, ^70^, ^89^ the TDR program. These eight independent samples were included in six meta‐analyses. Sensitivity analyses determined that there were no differences between the results of these six meta‐analyses when these independent samples were included in the meta‐analyses, in comparison to when they were excluded (data not shown).

## 4 DISCUSSION

This systematic review with meta‐analyses demonstrated that for adults with a BMI greater than or equal to 25 kg/m^2^, TDR programs had no clear adverse effect on mental well‐being. In fact, improvements were observed for several sub‐domains of mental well‐being, namely, depression, anxiety, stress, vitality, role‐emotional, and social functioning. These improvements were seen by post‐diet (0 to 2 months after the TDR program), and the improvements for depression, vitality and role‐emotional were maintained at follow‐up (2 months to 8 years after the TDR program). These results are particularly encouraging given that improvements in mental well‐being are not the primary focus of TDR interventions. While 22 of our 24 meta‐analyses assessed change in mental well‐being over time in TDR programs without comparators (due to a lack of mental well‐being research comparing TDR programs to a comparator), we found no clear evidence that TDR programs result in marked adverse effects on mental well‐being.

Researchers have excluded participants presenting with psychiatric symptoms from weight loss trials.^30^, ^55^, ^99^ In our systematic review with meta‐analyses, 60.0% (21/35) of publications reporting on 34 trials excluded participants with either a history of, or current, psychiatric symptoms.^29^, ^30^, ^31^, ^55^, ^56^, ^67^, ^69^, ^70^, ^71^, ^72^, ^75^, ^79^, ^81^, ^82^, ^83^, ^84^, ^85^, ^89^, ^91^, ^93^, ^100^ While a possible contributor to these exclusions is the potential impact of mental health conditions (and associated pharmacotherapy) on primary outcome variables (e.g., weight change), concerns around exacerbating mental health conditions^25^, ^26^ and lack of adherence to treatment protocols^27^ may be two additional reasons for these exclusions. While assessment of adherence to treatment protocols was beyond the scope of this systematic review, our meta‐analyses determined whether participants engaging in a TDR program experienced either an increase in depression, anxiety, stress, or negative affect scores, or a decrease in positive affect, mental quality of life, self‐esteem, or general psychological health scores. Despite the severely restrictive nature of these dietary programs, we saw no clear evidence that a TDR program results in marked adverse effects on mental well‐being. Additionally, while there is a lack of TDR research which directly compares those with existing mental health conditions to those without, all four of the meta‐analyses in this work that were amenable to subgroup analyses (depression pre‐diet to post‐diet; vitality pre‐diet to follow up; social functioning pre‐diet to follow‐up; and mental health pre‐diet to follow‐up) showed no difference between the pooled result of independent samples which excluded participants based on mental health factors and the pooled result of independent samples that did not exclude participants on this basis. This suggests that mental health status at pre‐diet does not have a unique effect on any of the post‐diet or follow‐up scores that were assessed in our subgroup analyses. In fact, improvements in all four of the above‐mentioned mental well‐being sub‐domains were found regardless of whether publications included or excluded participants based on mental health status. Taken together, we found no clear evidence to suggest that researchers should exclude participants from future trials involving TDR programs based on concerns that these diets may adversely affect mental well‐being. It is important to note, however, that the pre‐diet mean for all mental well‐being scales assessed in this review indicated low to moderate signs of illness, and as such, our results are unlikely to generalize to participants with severe mental health issues. Therefore, research into the use of TDR programs in people with moderate to severe mental illness would need to proceed with due caution.

Our results differ somewhat from previous meta‐analytic research examining the effects of VLEDs (primarily administered via TDR programs) on affect.^40^ For instance, while both our results and that of a previous meta‐analysis^40^ found a significant reduction in depression scores (i.e., an improvement in mental well‐being) between pre‐diet and post‐diet, the previous meta‐analysis found that this improvement was contingent on several methodological or participant‐related factors.^40^ That is, VLED protocols which were longer in duration (at least 8  weeks), included a low‐intensity exercise regimen (as opposed to no exercise regimen), involved behavior therapy, and produced a total weight loss of at least 14.1 kilograms, were all independently associated with an improvement in depression.^40^ While none of these factors were found to influence the effect of a TDR program on depression scores in our results, we did find that the reduction in depression scores from pre‐diet to post‐diet was associated with the year of publication of the study, whereby earlier publications were more likely to exhibit higher effect sizes, indicating greater reductions in depression scores, than more recent publications. These results are interesting given that there was no significant relationship between year of publication and pre‐diet scores, nor was there any significant effect of pre‐diet scores on improvement in depression pre‐diet to post‐diet. While improved experimental and/or reporting standards^101^ may be providing a more accurate representation of effect sizes in more recent publications, there was no relationship between *p* values and publication year, nor was there a difference between studies published prior to (or in/after) 2016, which was the year when the Clinical Trial Regulation requirements were instituted with the aim of improving experimental and/or reporting standards for clinical trials.^98^ Taken together, we found no clear reason for the significant effect of publication year on the observed improvement in depression scores from pre‐diet to post‐diet. In fact, we found that despite the improved experimental and/or reporting standards of recent years,^101^ the observed reduction in depression scores between pre‐diet to post‐diet remains clear and significant. For anxiety, our finding of significant reductions in anxiety scores from pre‐diet to post‐diet are also contrary to the previous meta‐analysis, which reported no effect of VLEDs on anxiety.^40^ There were, however, several methodological differences between the previously‐reported meta‐analytic findings and our meta‐analyses which may account for the differences in results for depression and anxiety. First, as we assessed a broader range of databases using a more comprehensive list of search terms, we found approximately twice as many independent samples to include in both our depression pre‐diet to post‐diet, and anxiety pre‐diet to post‐diet meta‐analyses. This, consequently, yielded greater power for both our depression and anxiety pre‐diet to post‐diet analyses than that of previous meta‐analytic research.^40^ For our meta‐analysis of depression pre‐diet to post‐diet, this increased power may have increased its overall resistance to changes in methodology, suggesting that improvement in depression with a TDR program is stronger than previously reported. Additionally, for anxiety pre‐diet to post‐diet, the increased power in our current meta‐analyses, in comparison with the previously‐published meta‐analysis,^40^ improved the likelihood of finding an effect if present. A second possible reason for the difference between our meta‐analysis results and those of previous meta‐analytic research is the differences in inclusion criteria.^40^ The previous publication,^40^ included studies which: evaluated all types of VLED diets (not only predominately liquid TDR programs but also those that were food‐based in nature); allowed for all participant pre‐diet weights, and thus included one publication with participants of “normal weight” at pre‐diet, determined through BMI assessment^102^; assessed one study involving inpatients^103^; and included one study in which the pre‐diet measurement was taken 1 week after commencement of the diet.^104^ In reviewing the combined results of our study and previous research,^40^ health care providers can be fairly confident that VLEDs and TDR programs are unlikely to cause a marked increase in depression or anxiety scores, and can, in fact, be hopeful that these diets may actually reduce mood‐related symptomatology. Our prediction intervals not only support this conclusion, but also allow researchers to predict these prospective effects with a certain level of accuracy. Here, we found that by follow‐up, mean depression scores are only likely to increase in less than 3% of future TDR interventions which meet our inclusion/exclusion criteria. For anxiety, mean scores are only likely to increase in less than 20% of future TDR interventions at follow‐up. It is also important to note that while increases in mean depression or mean anxiety scores may be observed in some future trials, it is unclear whether these increases will represent a significant change from mean pre‐diet levels.

Our meta‐analyses also determined a clear reduction in stress between pre‐diet and post‐diet.^93^ Our prediction interval for stress found that only 0.48% of future trials examining stress are likely to report a mean increase in stress levels between pre‐diet and post‐diet. To our knowledge, this is the first systematic review with meta‐analyses to examine the effects of TDR programs on stress. While research examining the unique effects of TDR programs on stress is in its infancy, these results are encouraging for future TDR trials.

In addition to reductions in depression, anxiety, and stress, there were also some noteworthy improvements across some of the mental quality of life sub‐domains. For instance, for vitality (i.e., a feeling of positive energy and strength), improvements from pre‐diet to post‐diet and from pre‐diet to follow‐up were clear and consistent. While previous research has indicated vitality is responsive to weight loss produced through moderately energy‐restricted food‐based diets,^105^ our results demonstrate that weight loss achieved through a severely energy‐restricted TDR program can also improve participant vitality from pre‐diet at both post‐diet and follow‐up. Our prediction intervals indicated that less than 4.0% of future TDR interventions which meet the inclusion/exclusion criteria in this review, are likely to produce a decrease in mean vitality scores by follow‐up.

Our subgroup analyses for vitality found that while independent samples with an average weight loss of less than 5 kilograms at follow‐up showed no improvements in vitality scores, independent samples with an average weight loss of 5 kilograms or more at follow‐up showed improvements in vitality scores. This result may be partially accounted for by two samples included in this subgroup analysis which reported that improvements in vitality were only observed for participants who lost more than 10% of their body weight at follow‐up.^81^, ^92^ While measuring percentage‐based changes in mental well‐being was beyond the scope of this review, both our findings and that of previous research suggest that for participants in trials of TDR programs, weight loss maintenance at follow‐up may be a necessary component for increased vitality scores to be observed.^81^, ^92^ It is important to note, however, that our meta‐regression did not find a direct relationship between the amount of weight change and the level of improvement in vitality scores between pre‐diet and follow‐up. Our subgroup analyses also found that trials which involved participants with a mean pre‐diet age younger than 45 years reported improved vitality scores at follow‐up, whereas trials involving participants with a mean pre‐diet age of 45 years or older reported no improvements in vitality scores. These findings for vitality relating to age and weight loss may be the result of a differing pattern of compliance across age and weight loss groups, whereby participants who were younger than 45 years at pre‐diet and who had lost more than 5 kilograms at follow‐up may have been more compliant with the diet. Previous research examining the effect of age on compliance and weight loss in a TDR program is mixed, with some authors reporting an increased dropout rate for participants aged less than 40 years,^106^ others reporting less weight loss in older participants,^107^ and recent trials reporting no difference in age between completers and non‐completers.^108^ While compliance was not examined in the review, future researchers could benefit from exploring the potential interplay between vitality, weight loss, and age to further understand these relationships.

For role‐emotional, we found that scores following the TDR program were improved from pre‐diet at both post‐diet and follow‐up, suggesting that participants were less limited by emotional factors, and were thus better able to engage in their work and other activities than before the diet. However, the effect size (pooled SMD estimate) of this emotional shift in engagement was small. The take‐home message from these findings is that a TDR program is unlikely to lead to a marked adverse effect on role‐emotional scores. In fact, our prediction intervals showed that less than 16.0% of future TDR interventions are likely to produce a decrease in mean role‐emotional scores from pre‐diet levels. Additionally, for role‐emotional pre‐diet to follow‐up, we found that independent samples which comprised participants with a mean pre‐diet BMI of 40 kg/m^2^ or more showed no significant change in role‐emotional scores from pre‐diet to follow‐up, whereas significant improvements in role‐emotional scores were observed between pre‐diet and follow‐up for samples which comprised participants with a mean pre‐diet BMI under 40 kg/m^2^. This finding may be due to the increased co‐morbidities experienced by those with a higher BMI^109^, ^110^ and that these co‐morbidities may exhibit an overarching effect on a participant's ability to engage in their everyday roles, over and above any benefits which may arise from TDR trial participation.

For mental health, our subgroup analyses found that from pre‐diet to follow‐up, the inclusion (or not) of psychological/cognitive counseling exhibited a paradoxical effect on pooled SMD estimates, whereby independent samples which included psychological/cognitive counseling showed no difference in mental health scores between pre‐diet to follow‐up, whereas independent samples which did not include psychological/cognitive counseling showed a significant improvement in scores. While the reasons underlying this finding may be related to greater expectations of mental health improvements among those participants which were provided with psychological/cognitive counseling,^111^ we found no evidence of similar results across other factors assessed in this review, and previous meta‐analytic research exploring the application of psychological/cognitive counseling has found that not only can counseling increase weight loss,^112^ but it can also improve psychological outcomes in weight loss interventions.^113^

We also found that less than 15.0% of future TDR interventions are likely to produce a reduction in mean social functioning scores. It may sound counterintuitive that a TDR program could improve social functioning, given recent evidence suggesting that participants engaging in these diets tend to avoid social situations as a means of allaying social and other pressures to consume foods not in accordance with the dietary program.^114^ It is important to remember, however, that post‐diet data for this review was collected up to and including 2 months (8.7 weeks) after the end of the diet, by which time participants were consuming one or more food‐based meals per day. Interestingly, our prediction interval indicated that 37.86% of future trials of TDR programs matching our inclusion/exclusion criteria may report a decrease in social functioning between pre‐diet and follow‐up. While the potential reasons for this decrease were beyond the scope of this review, it might be that participants on TDR programs may avoid food‐based social gatherings out of concerns about potential weight regain. Given this finding, we suggest that future researchers could do well to encourage adequate social support of participants on TDR programs, and where possible, provide the appropriate resources to manage any decline in social functioning.

While the results of our meta‐analyses of self‐esteem found that there were no significant shifts in self‐esteem scores from pre‐diet to post‐diet, or from pre‐diet to follow‐up, our prediction intervals determined that from pre‐diet to post‐diet, 65.2% of future interventions meeting our inclusion/exclusion criteria may show a decrease in mean self‐esteem scores, albeit the degree of any potential decrease cannot be ascertained from current data. These results differ from previous systematic and meta‐analytic research which found consistent improvements in self‐esteem with weight loss interventions involving either dietary and/or behavioral means, albeit prediction intervals were not included in the review of those publications.^105^ While it is possible that participants included in our meta‐analysis of self‐esteem from pre‐diet to post‐diet may have experienced unwanted side effects of rapid weight loss (e.g., sagging skin),^115^ and/or that the resulting weight loss may not have provided the improvements in confidence that participants were anticipating, it seems unlikely that these effects would be limited to our results for self‐esteem. That is, we would have expected similar effects in the other mental well‐being scales (e.g., depression and anxiety) rather than our results demonstrating clear improvements in these sub‐domains. One potential reason for our findings for self‐esteem may be the lower power of the meta‐analysis of self‐esteem from pre‐diet to post‐diet (4 independent samples with 152 participants) and/or that all independent samples in this meta‐analysis were drawn from one publication,^54^ suggesting that the external validity of these results (i.e., the ability to draw conclusions about wider populations due to improved representativeness) is questionable. In any case, future researchers could do well from monitoring participants' self‐esteem, and also managing participants' expectations regarding life following the TDR program.

The above‐mentioned improvements noted across several mental well‐being sub‐domains may be partially explained by diet‐specific and biological factors. First, while the nature of a TDR program is highly restrictive, these diets are beneficial in that the burden of food preparation and portioning is removed, thus creating a simple dietary structure for participants to adhere to. As for biological factors, changes in activity of the hypothalamo‐pituitary‐adrenal axis and/or serotonin system as well as the production of endogenous endorphins and ketones are likely to occur within the first few weeks on a TDR program due to the severe energy restriction.^116^ It is possible that the improvements in mental well‐being seen at mid‐diet and potentially at post‐diet, may be—in part—a result of these biological shifts.^116^ It is also important to note that physical quality of life (e.g., physical functioning) is likely to have improved across participants included in our meta‐analyses,^117^, ^118^ and that any improvements in physical quality of life may have had indirect effects on mental well‐being.

The main strength of our review was the use of broad search parameters, implemented across six databases, which yielded 25,976 screenable publications. These comprehensive search parameters allowed us to understand how TDR programs affect a broad range of mental well‐being sub‐domains, and as more independent samples were available for inclusion, the power of each meta‐analysis was increased, albeit not for all mental well‐being sub‐domains. Another strength of this review was that we calculated 95% prediction intervals for all meta‐analyses which contained more than two independent samples. These prediction intervals can help practitioners predict the range within which the mean of the true effects may fall for 95% of future populations and interventions meeting our inclusion/exclusion criteria. These prediction intervals help practitioners apply our results to future populations, with an increased level of confidence that is not afforded by meta‐analytic statistics alone (i.e., SMD and confidence interval data). Additionally, as we were provided with several participant‐level data sets from the authors of some of the included publications,^54^, ^55^, ^56^ we were able to impute missing data and analyze these datasets as intention‐to‐treat.

In addition to the above strengths, there were, however, several noteworthy limitations of our review. First, while we attempted to minimize bias from missing data at follow‐up by imputing some of our data from raw data sets provided by authors, as mentioned above, most of our included data were drawn directly from publications and a completers‐only analysis was used for most of our data as a conservative estimate rather than intention‐to‐treat. This completers‐only analysis does not account for bias resulting from dropouts, and therefore, it is possible that participants with improved mental and/or physical health outcomes are more likely to return for follow‐up assessment. Thus, the positive effects of TDR programs may be inflated in the publications included in our meta‐analyses. Second, while we devised a protocol regarding the review rationale, search strategy and inclusion/exclusion criteria, this protocol was not published, and thus the decisions made by the research team are not as transparent as they otherwise would have been. Third, due in part to the difficulties inherent in recruiting participants for a non‐active control, there is a scarcity of research on dietary obesity treatments using randomized controlled trials with a non‐intervention arm. Thus, for 22 of our 24 meta‐analyses, we could only explore single independent samples with no comparator group. This limits our ability to determine which effects are due to natural change over time, and which effects are due to the TDR intervention. However, across all 22 meta‐analyses without comparators, no marked adverse effects on mental well‐being were observed. With the comprehensive search and analysis structure of this review, we can be more confident that TDR programs are unlikely to result in marked adverse effects on mental well‐being, albeit more research needs to be conducted with comparators to clarify further. Fourth, due to the nature of trials of dietary interventions, researchers often employ strict inclusion/exclusion criteria to limit the influence of extraneous variables on the outcomes, and protect potentially vulnerable participants. Although this strategy is necessary for some participants, and helps researchers to identify the effect of the intervention on outcomes with greater clarity, thus improving the internal validity of the research, using strict inclusion/exclusion criteria prevents the inclusion of a diverse spectrum of participants, thus limiting the external validity of the study. By using these strict inclusion/exclusion criteria (e.g., no signs of depression or other mental illnesses), researchers created a group of participants which, while they may have a BMI in the overweight or obese category, are generally “psychologically healthy” with limited co‐morbidities. With more research supporting the safety and efficacy of TDR programs, future researchers can relax these strict inclusion/exclusion criteria and focus on improving the generalizability of their findings. A fifth limitation of the current review is that, as with many dietary trials, the majority of participants in the independent samples included in this review were women. Future researchers, therefore, should be careful when generalizing these results to adult males, as well as to those cohorts that would have been excluded from this review by virtue of our inclusion/exclusion criteria. One final noteworthy limitation of our review was that several of our analyses had a low number of independent samples and participants. In fact, 15/24 (62.5%) of our meta‐analyses included 5 independent samples or less with a median of 184 participants across these 15 meta‐analyses. These meta‐analyses may have lacked the power to determine whether a significant change in mental well‐being occurred in association with participation in a TDR program.

Taken together, our systematic review and meta‐analyses demonstrate that TDR programs are unlikely to result in marked adverse effects on mental well‐being in adults with a BMI greater than or equal to 25 kg/m^2^. That is, we found that TDR programs did not lead to significant increases in depression, anxiety, stress, or negative affect scores, and did not lead to significant decreases in positive affect, mental quality of life, self‐esteem, or general psychological health scores. In fact, depression, anxiety and stress scores decreased, and vitality, role‐emotional, and social functioning scores increased, suggesting improvements in these sub‐domains of mental well‐being. While two of our meta‐analyses were conducted with comparator groups, most were conducted without comparator groups, and thus it is important to recognize that other uncontrolled factors might be at play. Overall though, our results are encouraging considering no pooled negative effects on mental well‐being were observed across 24 meta‐analyses examining a wide variety of mental well‐being sub‐domains. These results are particularly striking given that the primary goal of TDR interventions is to reduce weight and improve cardiometabolic health and that mental well‐being—while important—is generally a secondary outcome. Also encouraging was our finding that improvements in depression from pre‐diet to post‐diet were not contingent on the inclusion of psychological/cognitive counseling, nor on the inclusion of behavioral/nutritional counseling in the TDR program. While this finding suggests that the TDR program alone may be capable of producing improvements in depression, clients with co‐morbid obesity and mental health concerns may require additional targeted treatment (e.g., psychotherapy and/or pharmacotherapy) in conjunction with the TDR intervention for optimal outcomes.

Given that our findings suggest that TDR programs are unlikely to result in marked adverse effects on mental well‐being, clinicians and researchers can begin to include participants across the mental health spectrum in TDR programs without undue concerns that these diets may cause psychological harm, albeit individual responses can vary. As obesity and its adverse consequences become increasingly prevalent, it is important to ensure that effective treatments are safe and accessible. Reducing the barriers which prevent people from engagement in TDR programs will help to foster greater inclusivity in healthcare environments.

## CONFLICTS OF INTEREST

A. Sainsbury reported owning 50% of the shares in Zuman International Pty Ltd, which receives royalties for books she has written and payments for presentations at industry conferences; receiving presentation fees and travel reimbursements from Eli Lilly and Co, the Pharmacy Guild of Australia, Novo Nordisk, the Dietitians Association of Australia, Shoalhaven Family Medical Centres, the Pharmaceutical Society of Australia, and Metagenics; and serving on the Nestlé Health Science Optifast VLCD advisory board from 2016 to 2018. R.V. Seimon reported serving on the Nestlé Health Science Optifast VLCD advisory board. A.A. Gibson reported receiving payment from the Pharmacy Guild of Australia and from Nestlé Health Science for presentations at conferences. H.A. Fernando and R.A. Harris reported being employed by the University of Sydney as tutors.

## ROLE OF THE FUNDER/SPONSOR

These funding bodies had no role in the design of this review; nor the collection, management, analysis, and interpretation of the data; preparation, review, approval of the manuscript; nor the decision to submit the manuscript for publication.
